# Novel Nitroxide-Substituted Hydrazone Switch: Experimental and Theoretical Insights into Photoswitching Behavior

**DOI:** 10.1021/acsorginorgau.5c00068

**Published:** 2025-12-03

**Authors:** Lucie Kotásková, Ivan Nemec, Radovan Herchel, Vinicius T. Santana, Petr Neugebauer

**Affiliations:** † 232848 Central European Institute of Technology, Brno University of Technology, Purkyňova 656/123, 61200 Brno, Czech Republic; ‡ Department of Inorganic Chemistry, Faculty of Science, 48207 Palacký University, 17. listopadu 12, 77900 Olomouc, Czech Republic

**Keywords:** hydrazone switch, nitroxide radical, photoisomerization, pH-induced isomerization, computational studies

## Abstract

Hydrazones are a versatile class of molecular switches with dual responsiveness to both light and pH. To investigate their switching properties, we incorporated a nitroxide moiety, enabling analysis by not only conventional techniques such as ^1^H NMR and UV–vis spectroscopy but also EPR spectroscopy, which provides valuable insights into structure and dynamics. A novel nitroxide-substituted hydrazone switch (**2**) was synthesized and fully characterized. However, initial experiments using ^1^H NMR and UV–vis revealed restricted photoisomerization of **2**. Theoretical studies employing DFT and TD-DFT methods revealed the presence of the D_1_ excited state related to π → π* electron transfer of the nitroxide moiety, and D_2_ excited state related to π → π* electron transfer within the hydrazone moiety. The latter excitation results in weakening of the C=N bond and enables the rotation around the hydrazone bond; however, the internal conversion D_2_ → D_1_ process is most likely responsible for the quenching of photoisomerization in **2**. Additionally, pH-induced switching was monitored using UV–vis and EPR spectroscopy, revealing that strong acids such as trifluoroacetic acid had no significant effect on the paramagnetic center.

## Graphical abstract

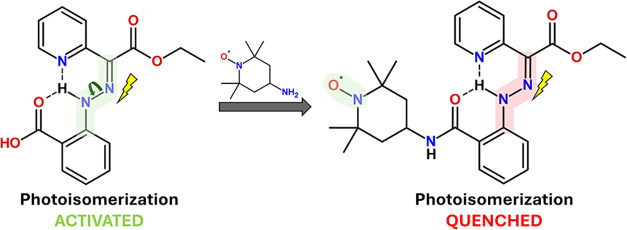

The straightforward formation and hydrolytic stability of the hydrazone functional group (C=N–NH) make these molecules advantageous for numerous applications.^1^ Due to the nucleophilic and electrophilic characteristics of the carbon atom, along with the nucleophilic properties of the nitrogen atom, hydrazones are suitable candidates for sensing applications involving both cations^2^ and anions.^3^ Their antimicrobial activity,^4^ as well as their role as agents in organocatalysis,^5^ represents another important area where hydrazones play crucial roles.

A key feature of the hydrazone group is its ability to undergo *Z*, *E* isomerization, resulting in the formation of two stable isomers that retain distinct properties.^1^ This characteristic has made hydrazones highly attractive in the field of molecular switches, molecules that can exist in two or more different, thermodynamically stable states and can be shifted between these states in response to an external stimulus. Hydrazones hold a prominent position among molecular switches due to their responsiveness to both light^6^ and pH changes.^7^ The dynamic nature of the hydrazone C=N–NH group, which allows for conformational, configurational, and constitutional flexibility, makes hydrazones widely used as building blocks in supramolecular architectures.^8^

In photomolecular applications, their appeal lies in a range of favorable properties, including high thermal half-lives, efficient photoconversion, resistance to photofatigue, tunable absorption wavelength, the ability to switch in various media, and the capacity to modulate fluorescence emission.^9,10^ As a result, hydrazones have been employed in diverse applications, including emissive hydrogels,^11^ drug delivery systems,^12^ molecular actuators,^13^ and molecular solar thermal energy storage (MOST) devices.^14^

Beyond their fundamental switching properties, recent studies have explored combinations of hydrazone switches with a paramagnetic species. For example, when functionalized with macrocycle that coordinates metal ions such as Gd­(III) or Fe­(III), the hydrazone switch can induce changes in relaxation times upon *Z,E* isomerization, a property particularly relevant for magnetic resonance imaging (MRI).^15^ For studying paramagnetic species, electron paramagnetic resonance (EPR) can be recognized as a suitable tool, but the reports on studying hydrazone switches by EPR remain largely unexplored. This is primarily due to the scarcity of reports on radical-functionalized hydrazones.

Only a few studies reported the combination of hydrazone and nitroxide radicals within a single molecule. For example, a spin-labeled derivative of podophyllotoxin containing a hydrazone group demonstrated antitumor and antioxidant activity. In this study, the importance lies in the antioxidant properties of the nitroxide moiety, while the hydrazone group served merely as a linker between nitroxide and podophyllic acid.^16^ Another study described a pH-responsive theranostic system in which doxorubicin, attached to nitroxide through a polymeric backbone via a hydrazone linker, is released upon acidification and subsequent hydrolysis of the hydrazone bond. Concurrently, the fluorescence, which was previously quenched by the nitroxide, is restored, allowing for the quantification of doxorubicin release.^17^

EPR spectroscopy plays a crucial role in analyzing complex supramolecular structures labeled with a paramagnetic species. It provides high sensitivity and allows for the extraction of kinetic information in the submicrosecond time range, as well as measurements of molecular tumbling rates on the nanosecond time scale. Moreover, pulsed electron–electron double resonance (PELDOR) spectroscopy provides distance measurements up to 100 Å, serving as an essential tool for studying various conformations.^18^ Nitroxides are frequently used as spin labels,^19^ or spin probes,^20^ to facilitate the investigation of the structure and dynamics of supramolecular assemblies.^21–23^ The attachment of nitroxide radicals to simpler systems with switching behavior, such as overcrowded alkenes^24^ or azobenzenes,^25^ has been reported; however, to our knowledge, spin-labeling of hydrazone switches has not yet been explored.

The nature of the hydrazone rotor plays a critical role in the photoisomerization efficiency. Although pyridine rotor-based hydrazones exhibit good switching properties in response to pH change due to the basic nitrogen in the pyridine ring, their photoswitching capabilities are considerably limited. The *Z*, *E* isomerization is hindered by strong intramolecular hydrogen bonding between the pyridine nitrogen and the hydrogen atom of the hydrazone group (N···H–N). A significant improvement was achieved by replacing the pyridine ring with a phenyl ring in the rotor.^6^ However, we recently demonstrated that *ortho*-halogen substituted hydrazones with pyridine rotors can undergo *Z*, *E* isomerization upon irradiation at 420 nm and reverse *E*, *Z* isomerization at 460 nm.^26^ The photoswitching mechanism was clarified through theoretical calculations, which revealed the formation of a new diastereomer upon *E*, *Z* isomerization, a phenomenon observed for the first time to our knowledge. Additionally, we investigated the effects of different halogen atoms on photoswitching properties and thermodynamic parameters, showing that these *ortho*-halogen substituted switches could be useful in MOST applications due to their sufficiently high activation energy barriers.^26^

Building on the promising results of photoswitchable pyridine rotor-based hydrazones, we introduce a new *ortho*-nitroxide-substituted hydrazone switch incorporating a pyridine rotor. The aim of this study is to investigate the photoswitching properties of this nitroxide-substituted hydrazone switch (**2**) through both experimental and theoretical approaches. Our findings reveal that the carboxylic hydrazone precursor (**1**) exhibits partial and reversible photoisomerization, whereas its radical analogue (**2**) shows restricted photoisomerization. The incorporation of the nitroxide radical offers new opportunities to investigate the dynamics of the hydrazone system using EPR spectroscopy. The hydrazone group’s responsiveness to light or pH changes suggests potential for developing novel adaptive probes.

## Experimental Section

### Materials

All chemicals were purchased from commercial suppliers and used without further purification. Dichloromethane (DCM) was dried by pouring over activated molecular sieves (3Å) under an argon atmosphere. Purifications using column chromatography were performed using 60–200 μm silica gel 60. The reaction progress was monitored by a TLC on precoated aluminum sheets and visualized using UV lamp (254 and 365 nm). Ethyl (pyridin-2-yl)­acetate was synthesized using reaction conditions from previously published work.^26^

### Instrumentation

^1^H and ^13^C­{^1^H} NMR spectra were recorded on a Bruker spectrometer operating at 700 MHz (Bruker Avance Neo) using DMSO-*d*_6_ as solvent. NMR spectra interpretation was performed using Bruker TopSpin software.^27^ Chemical shifts on the δ scale (ppm) relative to tetramethylsilane were referenced internally with respect to either the protio resonance of residual DMSO-*d*_6_ (δ_H_ = 2.50 ppm and δ_C_ = 39.52 ppm). X-band EPR measurements were recorded using an EPR spectrometer Bruker EMX Nano with magnetic field modulation amplitude of 0.5 G at 100 kHz, 10 mW of microwave power, and 50 s scans. The EPR spectra were simulated using EasySpin toolbox.^28^ For EPR measurements, compound **2** was dissolved in toluene (Rotisolv HPLC) that was previously degassed by constant nitrogen flow for an hour to reduce oxygen content. UV–vis spectra were recorded using a Jasco V-770 UV/vis/NIR spectrophotometer equipped with a single monochromator and deuterium and tungsten-halogen lamps, covering a wavelength range of 190–2700 nm. Samples were placed in 10 mm quartz cuvettes. Mass spectra were acquired on a Thermo Scientific LCQ Fleet mass spectrometer (Waltham, MA, USA) with an electrospray ionization source and a three-dimensional (3D) ion-trap detector. Spectra were recorded in positive mode over an *m*/*z* range of 50–2000, using the following parameters: spray voltage = 5 kV [−4.83 (−), 5.22 (+)]; capillary temperature = 275 °C; capillary voltage = 50 V; tube lens = 120 V (+), – 100 V (−). Photoswitching experiments were performed in DMSO or toluene using a custom irradiation chamber fitted with high-power LED diodes at 365, 420, or 460 nm. All experiments were carried out under refrigerated conditions to avoid overheating and prevent exposure to ambient light. For UV–vis measurements, samples were irradiated in standard 10 mm quartz cuvettes, while 3 mm and 5 mm tubes were used for EPR and NMR experiments, respectively. Samples were exposed to 365 or 420 nm light to induce *Z*, *E* isomerization, and spectra were recorded after transferring the samples from the irradiation chamber to the spectrometer, ensuring they remained protected from light during the transfer.

### Crystallography

The X-ray diffraction data (Table S1) collection for the selected single crystals was carried out using an XtaLAB Synergy-I diffractometer (Rigaku Corporation, Tokyo, Japan) equipped with a HyPix3000 hybrid pixel array detector and a microfocused PhotonJet-I X-ray source (Cu Kα) at 100.0(2) K for **1** and 90.0(2) K for **2**. Data integration, scaling, and absorption corrections were performed using CrysAlisPro 1.171.40.82a.^29^ The crystal structures were solved using SHELXT,^30^ and all nonhydrogen atoms were refined anisotropically on F^2^ using the full-matrix least-squares method with Olex2.refine^31^ in OLEX2 (version 1.5).^32^ Hydrogen atoms were identified in differential Fourier maps, and their parameters were refined by using a riding model with *U*_iso_(H) = 1.2*U*_eq_ for −CH_2_– groups and 1.5 *U*_eq_ for −CH_3_ groups. Compound **1** was obtained as yellow crystals by the slow evaporation of an acetonitrile solution. Compound **2** was obtained as orange-brown crystals by the diffusion of *n*-heptane into a DCM solution of **2**.

#### Synthesis of 2-{(2*Z*)-2-[2-Ethoxy-2-oxo-1-(pyridin-2-yl)­ethylidene]­hydrazinyl}­benzoic Acid (**1**)

Anthranilic acid (0.61 mmol, 1 equiv) was dissolved in 0.6 mL H_2_O and 0.15 mL concentrated HCl and cooled to 0 °C. The aqueous solution of NaNO_2_ (0.67 mmol, 1.1 equiv) was added dropwise to the thoroughly stirred anthranilic hydrochloride. The diazonium salt was stirred for an additional 15 min at 0 °C. Ethyl (pyridin-2-yl)­acetate (0.61 mmol, 1 equiv) and CH_3_COONa (5.45 mmol, 9 equiv) were dissolved in a mixture of EtOH/H_2_O (5:1, v:v) and cooled to 0 °C. The diazonium salt was added dropwise to the mixture. Immediately, after the addition of the first portions of diazonium salt, the transparent mixture of ethyl (pyridin-2-yl)­acetate and CH_3_COONa became yellow. After the addition of whole portion of diazonium salt, the formation of bright yellow precipitate was observed. The mixture was stirred overnight at room temperature. The completion of the reaction was monitored by TLC. The precipitate was filtered, washed with cold H_2_O, and dried under vacuum yielding compound **1** as a bright yellow solid. Slow evaporation of an acetonitrile solution of **1** furnished yellow crystals. Yield: 167 mg, 88%, ^1^H NMR (700 MHz, DMSO-*d*_6_): δ 15.02 (s, 1H), 13.46 (br s, 1H), 8.75 (ddd, *J* = 4.8, 2.9, 2.1 Hz, 1H), 8.01 (td, *J* = 7.5, 1.8 Hz, 1H), 7.95–7.90 (m, 3H), 7.63 (ddd, *J* = 8.7, 6.6, 1.6 Hz, 1H), 7.50 (ddd, *J* = 7.5, 4.8, 1.3 Hz, 1H), 7.05 (ddd, *J* = 8.1, 6.7, 1.3 Hz, 1H), 4.33 (q, *J* = 7.1 Hz, 2H), 1.35 (t, *J* = 7.1 Hz, 3H). ^13^C­{^1^H} NMR (176 MHz, DMSO-*d*_6_): δ 168.3 (s, 1H), 164.8 (s, 1H), 150.3 (s, 1H), 147.5 (s, 1H), 145.0 (s, 1H), 137.4 (s, 1H), 136.8 (s, 1H), 134.3 (s, 1H), 131.4 (s, 1H), 129.6 (s, 1H), 124.3 (s, 1H), 123.5 (s, 1H), 121.1 (s, 1H), 114.2 (s, 1H), 60.8 (s, 1H), 14.2 (s, 1H). MS *m*/*z* (−): calcd for C_16_H_15_N_3_O_4_ 647.1861 [2×(M–H)+Na]^+^; found 647.0647.

#### Synthesis of Compound **2**

Dry DCM was added to 1,1′-carbonyldiimidazole (0.16 mmol, 1 equiv) in a predried Schlenk flask that had been flushed with argon. After cooling the solution to 0 °C, a dry DCM solution of compound **1** (0.16 mmol, 1 equiv) was introduced dropwise. The mixture became bright yellow and was stirred for 1 h at room temperature under an argon atmosphere, during which a white precipitate formed. Subsequently, 4-amino-TEMPO (0.16 mmol, 1 equiv) was added in one portion as a solid, causing the mixture to turn orange. Stirring was continued overnight at room temperature. TLC indicated the appearance of a new product. Ethyl acetate was then added, and the organic layer was washed with 10% HCl (3 × 20 mL) followed by brine. The aqueous layer was extracted with DCM, and this extract was combined with an ethyl acetate phase. The combined organic solution was dried over MgSO_4_, filtered, and concentrated under reduced pressure. The crude material was purified by silica gel column chromatography (98% DCM/2% MeOH), affording compound **2** as a bright yellow solid. Dissolving compound **2** in DCM and layering with *n*-heptane produced orange-brown crystals. Yield: 43 mg, 59%, *R*_F_ = 0.66 (DCM/MeOH 96/4), MS *m*/*z* (+): calcd for C_25_H_32_N_5_O_4_^•^ 489.2347 [M + Na]^+^; found 489.2311. X-band EPR spectrum (toluene, 293 K): triplet, *g*_avg_ = 2.0075; *A*_avg_ = 44.0 MHz.

## Results and Discussion

### Synthesis and Characterization

Compound **1** was prepared through a Japp–Klingemann reaction, in which deprotonated ethyl (pyridin-2-yl)­acetate undergoes nucleophilic addition to 2-carboxybenzene-1-diazonium chloride (Scheme 1). Pure compound **1** was obtained simply by washing it with H_2_O, eliminating the need for purification by column chromatography. Its successful isolation was verified by ^1^H and ^13^C­{^1^H} NMR spectroscopy (Figures S1 and S2). Formation of the C=N–N–H hydrazone linkage was evidenced by a resonance at 15.02 ppm in the ^1^H NMR spectrum, corresponding to the N–H_*(Z)*_ proton. As is typical for hydrazones, compound **1** was not isolated as a single isomer; therefore, an additional N–H_*(E)*_ signal appeared at 12.77 ppm. Integration of these N–H resonances provided an isomer ratio of 81% *Z*-form to 19% *E*-form. Mass spectrometry further supported the identity of compound **1**, showing its most intense peak as the [2×(M–H)+Na]^−^ ion (Figure S3).

**Scheme 1:**
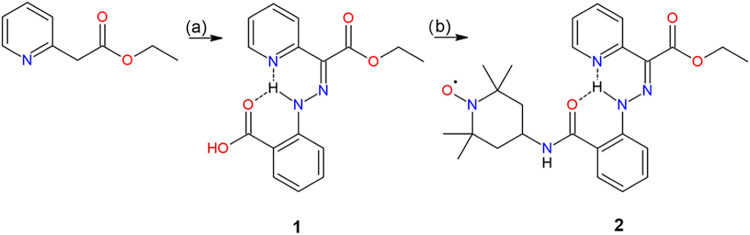
Reaction Scheme for the Preparation of Compounds **1** and **2** ^a^

a (a) i. anthranilic acid (1 equiv), NaNO_2_ (1.1 equiv), HCl, H_2_O, 0 °C; ii. ethyl­(pyridine-2-yl)­acetate (1 equiv), NaOAc (9 equiv), EtOH, H_2_O, 0 °C. (b) **1** (1 equiv), CDI (1 equiv), 4-amino-TEMPO (1 equiv), dry DCM, 0°C, Ar

The preparation of compound **2** began with the CDI-mediated acylation of 4-amino-TEMPO (Scheme 1). Initially, CDI activates the carboxylic acid function of compound **1**, generating an acyl imidazole intermediate and releasing imidazole; this was evident from the formation of a white precipitate after compound **1** was added to CDI. The acyl imidazole intermediate is then attacked by 4-amino-TEMPO, leading to amide bond formation. To maximize the overall yield, the workup procedure was refined. In the original protocol, the crude product was collected from the ethyl acetate layer after being washed with 10% HCl and brine. In the improved method, the aqueous phase obtained after these washings was additionally extracted with DCM, and this DCM extract was combined with the ethyl acetate fraction. Implementing this modification increased the reaction yield from 16 to 59%. After purification by column chromatography, compound **2** was obtained as a yellow solid and identified by MS and EPR analyses. The mass spectrum displayed a dominant peak at *m*/*z* 489.23 corresponding to the [M + Na]^+^ ion (Figure S4). EPR measurements of compound **2** (*c* = 45 μM) revealed a typical three-line signal arising from hyperfine coupling of the unpaired electron with a ^14^N nucleus (*I* = 1). Key EPR parameters derived from the spectral simulation are provided in Table S2.

### Crystal Structures

Compounds **1** and **2** were successfully crystallized into forms suitable for single-crystal X-ray diffraction analysis. Compound **1** crystallizes as yellow, needle-like monoclinic crystals (space group *P*2_1_/*n*, Table S1). In the solid state, it adopts exclusively the *E*-conformation (Figure 1), which is notable because the *Z*-isomer predominates in solution (*vide supra*). The *E*-form is stabilized through two intramolecular hydrogen bonds: one between the hydrazone N–H group and the carboxylic oxygen atom of the rotor (*d*(N···O) = 2.678(2) Å), and another between the same N–H group and the ethyl ester carbonyl oxygen of the stator (*d*(N···O) = 2.685(2) Å). Additionally, the carboxylic group participates in an intermolecular hydrogen bond with the pyridine nitrogen of a neighboring molecule (*d*(O···N) = 2.562(2) Å).

**Figure 1:**
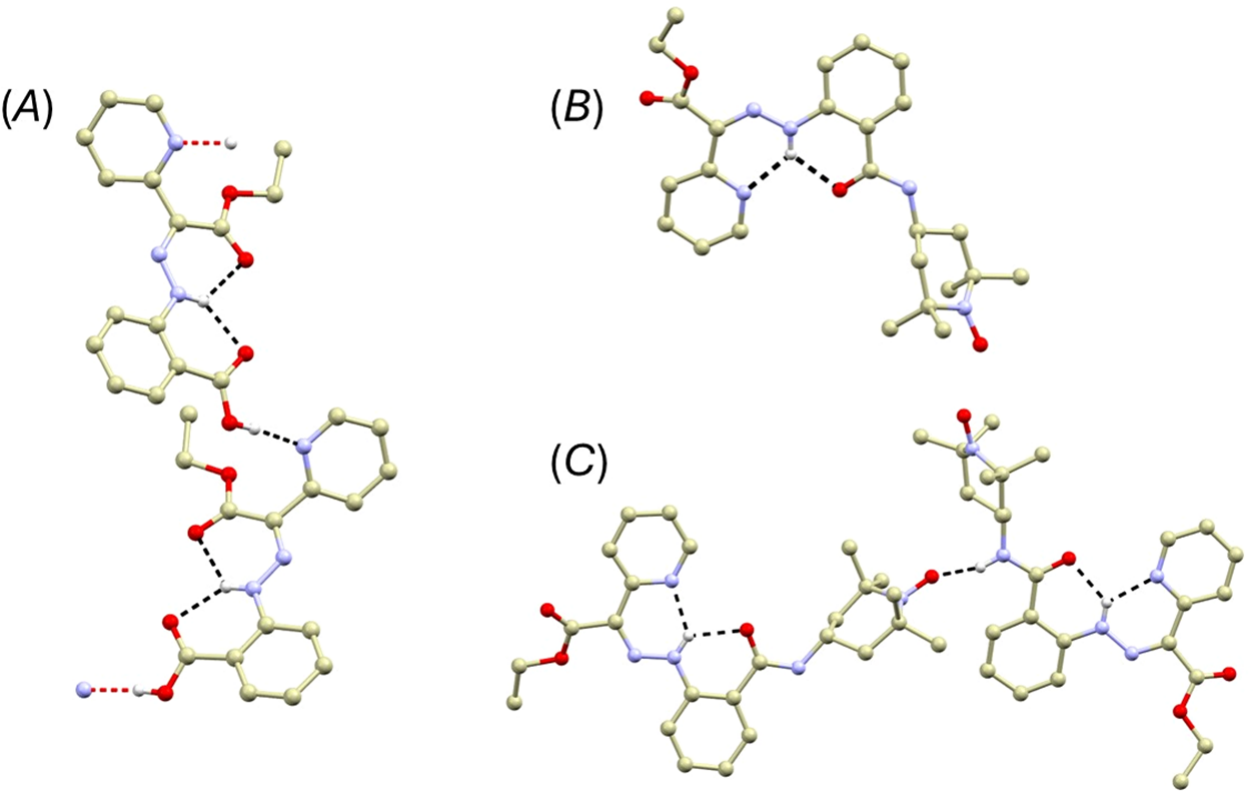
(A) Perspective view of a fragment of the crystal structure of compound **1**. (B) Molecular structure of compound **2**. (C) Perspective view of a fragment of the crystal structure of compound **2**. All hydrogen atoms except those involved in hydrogen bonding were omitted for clarity. Hydrogen bonds are represented by dashed lines: black dashed lines indicate standard hydrogen bonds, while red dashed lines represent hydrogen bonds that extend the crystal structure into the supramolecular polymer structure.

Compound **2**, in contrast, forms yellow prismatic monoclinic crystals (*P*2_1_/*c*, Table S1) and adopts exclusively the *Z*-conformation (Figure 1). Here, the hydrazone N–H group engages in bifurcated intramolecular hydrogen bonding with both the pyridine nitrogen of the rotor (*d*(N···N) = 2.622(3) Å) and the oxygen atom of the carboxamide function (*d*(N···O) = 2.642(3) Å). Furthermore, the carboxamide N–H hydrogen forms an intermolecular hydrogen bond with the oxygen atom of the adjacent TEMPO unit (*d*(N···O) = 2.792(3) Å).

In our earlier study on photoswitchable hydrazones bearing halide substituents on a pyridine stator, we found that intramolecular hydrogen bonding could significantly affect the photoswitching thermodynamics.^26^ Compounds **1** and **2** share the pyridine and ethyl carboxylate motifs observed previously but differ by possessing an additional carboxylic oxygen atom on the stator, which is capable of forming stronger hydrogen bonds with the hydrazone N–H group than halides. This prompted us to examine their intramolecular interactions through the Quantum Theory of Atoms in Molecules (QTAIM).^33^ Structural fragments from the experimental X-ray data were used, with hydrogen atom positions optimized via DFT calculations (r^2^SCAN-3c method) using the Orca 6.0 software suite.^34–42^ The resulting wave functions served as the basis for topological and energetic analyses (Tables S3 and S4) using Multiwfn^43,44^ and AIMAll^45^ software packages (Figure S6).

The analysis showed that intramolecular hydrogen bonds in compound **1** are weaker than those in compound **2**. The strongest interaction identified was the N–H···N hydrogen bond in **2**, with an interaction energy of 10.2 kcal·mol^–1^ (Table S4). The next strongest contact was the N–H···O contact involving the carboxamide oxygen in **2** (9.1 kcal·mol^–1^, Table S4). In compound **1**, the corresponding hydrogen bonds displayed slightly lower interaction energies of 7.8 and 8.2 kcal·mol^–1^ (Table S3).

### Photoisomerization

Photoswitching behavior of compound **1** was first examined by using ^1^H NMR and UV–vis spectroscopy. In DMSO-*d*_*6*_, the ^1^H NMR experiments demonstrated that compound **1** undergoes reversible photoisomerization upon irradiation at 420 and 365 nm (Figure S9). Photostationary states were reached after 10 min of irradiation at both wavelengths, giving *Z*/*E* ratios of 58/42 under 420 nm light and 78/22 under 365 nm light. UV–vis measurements recorded in toluene (Figure S10) revealed only minor spectral differences before irradiation (ε^379^ = 30,674 dm^3^·mol^–1^·cm^–1^) and after exposure to 420 nm (ε^371^ = 29,222 dm^3^·mol^–1^·cm^–1^), with absorption maxima (λ_max_) shifting by merely ∼8 nm. Photobleaching occurred during irradiation, and back-isomerization at 365 nm caused further intensity loss and shifted the absorption maximum to 374 nm (ε^374^ = 27,470 dm^3^·mol^–1^·cm^–1^). The initial UV–vis spectrum also contained a weaker absorption band at 340 nm that decreased upon 420 nm irradiation as the spectrum shifted hypsochromically. After subsequent back-isomerization at 365 nm, this sideband became more pronounced when the spectrum bathochromically shifted. This additional feature may correspond to the less stable *E*-isomer, which was present in an unusually high amount (17%) prior to irradiation.^6,10,26^ Given these findings, ^1^H NMR–monitored photoswitching experiments were next performed on compound **2** (Scheme 2). Its spectrum showed no detectable changes upon irradiation, even after extended exposure (2 h) at 420 nm (Figures 2 and S11). These observations clearly demonstrate that compound **2** exhibits strongly suppressed photoisomerization relative to its nonradical precursor **1**. Such suppression aligns with earlier results reported for ethyl-(2-phenylhydrazinylidene)­(pyridin-2-yl)­acetate,^6^ where intramolecular hydrogen bonding hindered photochemical isomerization. In the present case, not only the intramolecular N···H–N interaction but also an O···H–N contact (see Crystal Structures and Computational Studies) likely contributes to the reduced photoswitching efficiency.

**Figure 2:**
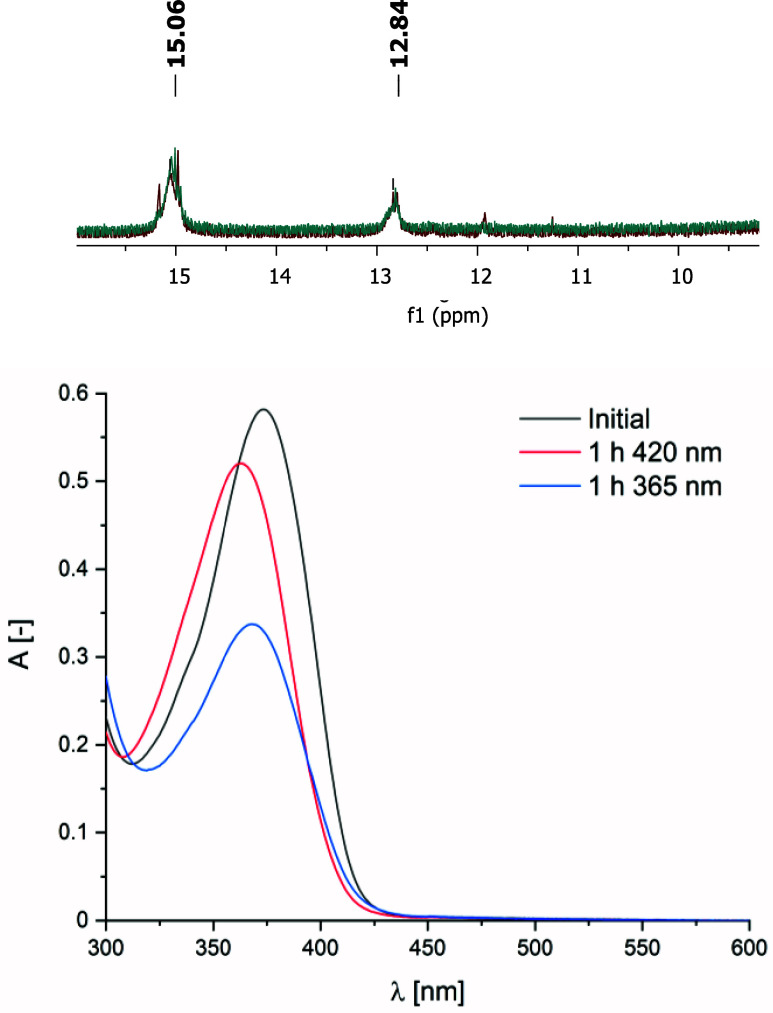
Top: Comparison of N–*H* resonances in ^1^H NMR spectra of compound **2** before (green) and after irradiation at 420 nm (red) for 2 h (DMSO-*d*_*6*_, 400 MHz, 298 K). Bottom: UV–vis spectrum of compound **2** (4.46 × 10^–5^ M, toluene) before and after irradiation at 420 nm for 1 h and 365 nm for 1 h.

**Scheme 2:**
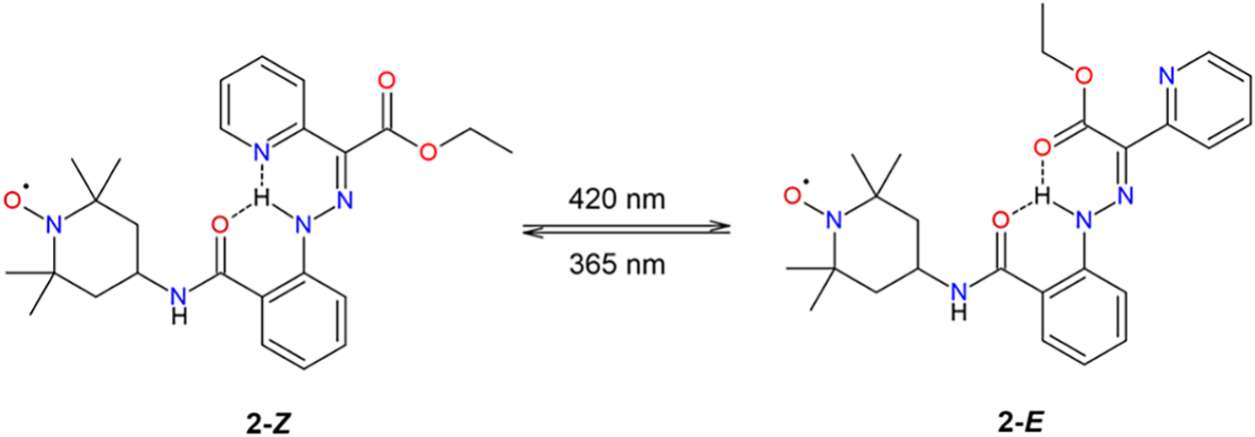
Considered Scheme for the *Z*, *E* Photoisomerization of Compound **2**

The photoswitching of compound **2** was further investigated using UV–vis spectroscopy (Figure 2), revealing behavior distinct from that of compound **1**. The initial UV–vis spectrum displayed a λ_max_ at 373 nm (ε^373^ = 13 048 dm^3^·mol^–1^·cm^–1^). After 1 h of irradiation at 420 nm, a hypsochromic shift was observed along with a decrease in absorption intensity (ε^363^ = 11 665 dm^3^·mol^–1^·cm^–1^). EPR spectroscopy conducted after 30 min of 420 nm irradiation (Figure S12) indicated that the paramagnetic center remained highly stable, which was corroborated by ^1^H NMR measurements (Figures 2 and S11). In contrast, irradiation at 365 nm for 1 h caused a pronounced decrease in stability, as evidenced by a significant drop in absorbance (ε^368^ = 7 563 dm^3^·mol^–1^·cm^–1^). EPR spectroscopy under the same conditions (Figure S13) confirmed this instability, showing a marked reduction in signal intensity compared with 420 nm irradiation. MS analysis of compound **2** after 365 nm irradiation revealed new peaks at *m*/*z* 452.17 and 474.22, corresponding to the decomposed secondary amine and its Na^+^ adduct, respectively (Figure S14). The reduced stability under UV–vis light is likely due to photodegradation, which can involve aminoxyl bond disproportionation into hydroxylamine and *N*-oxoammonium species, or bond cleavage leading to decomposition, dimerization, or rearrangements.^46^ Shorter irradiation times (1 min) at either 365 or 420 nm, however, did not affect the EPR signal intensity, indicating that radical concentrations were preserved (Figure S15). This effect was also observed at the higher sample concentrations required for NMR measurements, in contrast to EPR and UV–vis analyses. Irradiation of compound **2** (*c* ∼ 0.07 M) at 365 nm for 2 h did not result in any detectable decomposition, as the NMR signals before and after irradiation remained unchanged (Figure S16).

### pH-Induced Isomerization

Scheme 3 displays the expected mechanism of *Z*, *E* isomerization of compound **2** upon pH change. The UV–vis measurements showed characteristic bathochromic shift (by 32 nm) upon treatment with TFA (100 equiv) for compound **1** (Figure S17). The acid addition was accompanied by a characteristic color change in the sample, transitioning from light yellow to an intense yellow. Filtering the acidified solution through a plug of K_2_CO_3_ resulted in the solution returning to a pale-yellow color. This was accompanied by the return of the λ_max_ value to the initial value and lowering of the absorbance. A similar trend was observed for the pH switching of compound **2** (Figure 3). We already showed in our previous study that deprotonation with K_2_CO_3_ is a sensitive process, and its accomplishment highly influences the *A*_max_ value.^26^ The pH-induced switching of compound **2** was also monitored using ^1^H NMR, revealing the expected behavior (Figure S18). Protonation with TFA (2.6 equiv) induced rotation around the C=N bond, resulting in the *E*-isomer becoming the predominant form. This is evidenced by a downfield-shifted signal at 14.14 ppm corresponding to the N–*H*_(*E*)_ proton. Interestingly, the *Z*-isomer (15.09 ppm, N–*H*_(*Z*)_) was still present in a relatively high proportion (33% *Z*-isomer, 67% *E*-isomer). The signals in the spectrum retained their paramagnetically broadened character, indicating the integrity of the paramagnetic center in compound **2**. The addition of TFA was also accompanied by a broad signal in the 11–13 ppm range, likely attributable to the excess TFA protons; this signal disappeared upon deprotonation with K_2_CO_3_. Deprotonation restored the initial spectrum observed before protonation, confirming successful isomerization back to the initial state (75% *Z*-isomer, 25% *E*-isomer), consistent with the *Z*/*E* ratio of the unprotonated compound.

**Figure 3:**
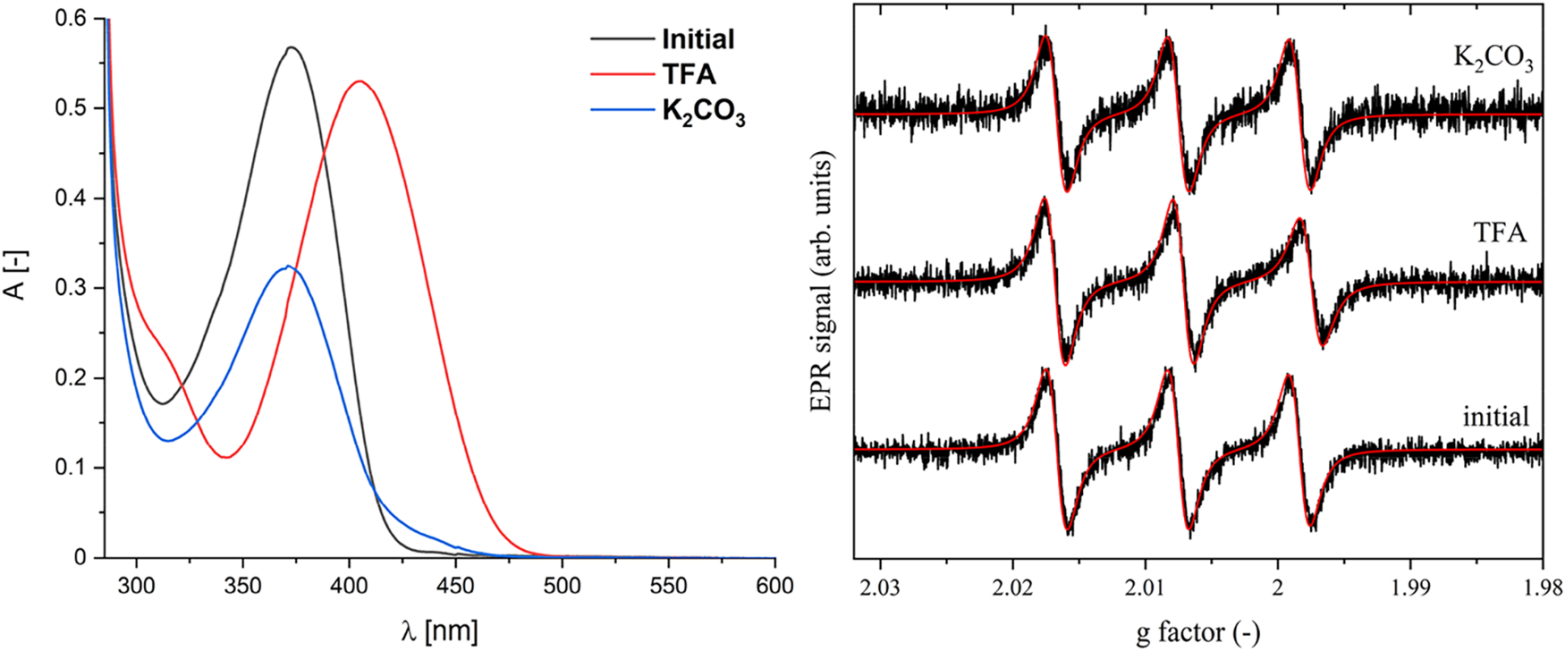
Left: UV–vis spectrum of compound **2** (4.6 × 10^–5^ M, toluene) before and after pH switching using trifluoroacetic acid (300 equiv) and filtering through the plug of K_2_CO_3_. Right: X-band EPR spectra of compound **2** before and after pH switching (using trifluoroacetic acid and K_2_CO_3_), 45 μM, toluene, 293 K.

**Scheme 3:**
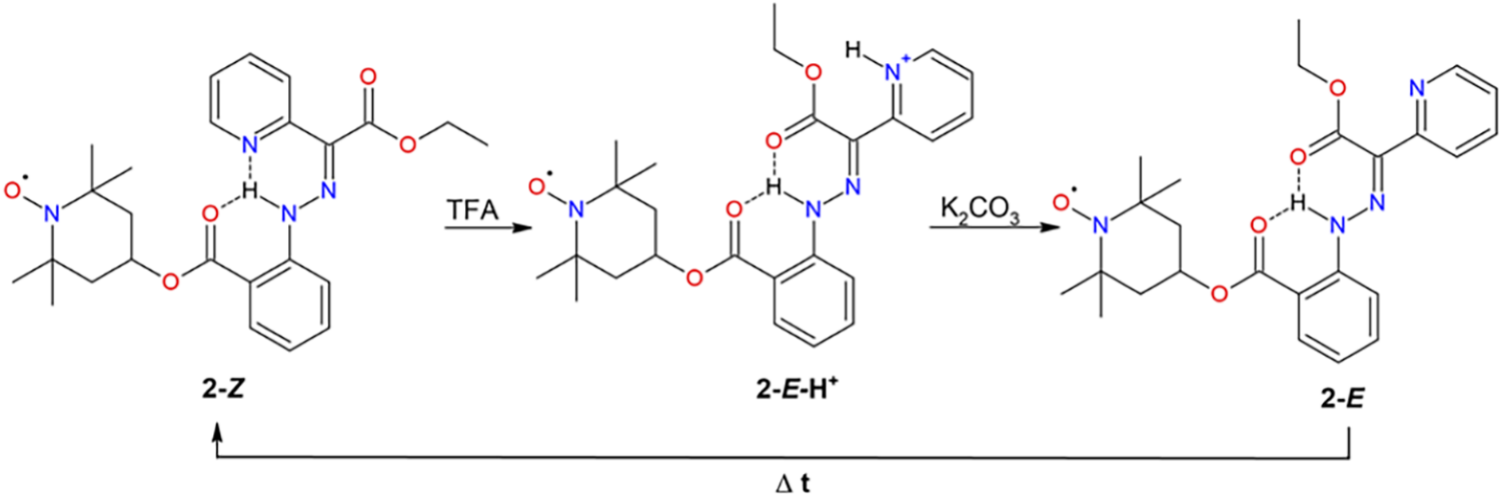
Expected Mechanism of *Z*, *E* Isomerization of Compound **2** upon pH Change (TFA = Trifluoroacetic Acid)

EPR spectroscopy was used to monitor pH-induced switching, revealing a pronounced decrease in the molecular mobility (Figure 3). The rotational correlation time (τ*_c_*) increased from 0.0405 to 0.19 ns, a 4.7-fold change, after the addition of trifluoroacetic acid (TFA), which also caused the intensity of the third signal in the EPR spectrum to decrease. Due to the low sample concentration (*c* = 45 μM), a 300-fold molar excess of TFA was added to ensure complete protonation. As expected, protonation of the pyridine nitrogen occurred, accompanied by the characteristic color change. Subsequent EPR measurements showed that the signal intensity was fully restored after treatment with K_2_CO_3_. These results indicate that the hypochromic effect observed in the UV–vis spectrum of compound **2** after deprotonation is not due to decomposition (Figure 3).

Interestingly, the τ*_c_* value decreased to 0.0212 ns, indicating partial recovery toward its original value (Table S2). The observed changes in τ*_c_* —an increase upon addition of TFA and a subsequent decrease following filtration through K_2_CO_3_—can be explained by differences in molecular mobility between the protonated form **2-** ***E*** **-H** ^**+**^ and the neutral, deprotonated species.

### Computational Studies

The photoswitching mechanism of hydrazones has already been elucidated using a combination of TD-DFT calculations, steady-state, and time-resolved spectroscopy.^47,48^ These studies revealed that after photoexcitation to the S_1_ state, rotation about the C=N and N–N bond is the preferred isomerization pathway, occurring on the S_1_ potential energy surface of the hydrazone. The excited molecule relaxes to an S_1_ global minimum with a C=N torsional angle of approximately 90.0°, reaching a conical intersection with the ground state. From this point, the molecule can either return to the initial ground-state geometry or proceed forward to form the metastable isomer. While in-plane nitrogen inversion is ruled out as a pathway for photochemical isomerization, it remains accessible for thermal isomerization due to its lower energy barrier in the ground state.^48^

The presence of intramolecular hydrogen bonding in hydrazones influences alternative photochemical isomerization mechanisms. Recently, an excited-state intramolecular proton transfer (ESIPT) mechanism was identified in the solid-state photochemical isomerization of perfluorinated hydrazones.^49^ In this system, the bulky hydrazine moiety restricted rotational motion due to constraints of the crystalline lattice. Instead, proton transfer occurred intramolecularly between a proton donor (−NH) and a proton acceptor (heteroatom) in the excited state, leading to pronounced photochromism that had not previously been observed in the solid-state switching of hydrazones.

Thus, the hydrazone compounds **1** and **2** were also investigated theoretically at the DFT and TD-DFT level of theory using ORCA 6.0/6.1 software.^34,50^ Similarly to our previous work on hydrazone switches,^26^ CAM-B3LYP range-separated hybrid functional was used^51^ together with the atom-pairwise dispersion correction (D4).^41^ The def2-TZVP basis set was used for all atoms.^52^ The calculations were speed-up using def2/J Coulomb fitting basis set^53^ and RIJCOSX approximation.^54^ The largest integration grid (DefGrid3) and tightSCF convergence criteria were used in all of the calculations. Additionally, the implicit solvation model C-PCM was employed for all calculations, using dichloromethane as the solvent.^55,56^ The calculated data were visualized with VESTA 3 program^57^ or with Chemcraft.^58^ The respective XYZ files of the computed molecular geometries are available in the Supporting Information. The transition states were also searched for using the Nudged Elastic Band method (NEB).^59^

To elucidate the distinct behavior of hydrazone compounds **1** and **2**, geometry optimization and energetics calculations were performed with CAM-B3LYP + D4/def2-TZVP/C-PCM­(CH_2_Cl_2_) for the two reaction mechanisms depicted in Scheme 4.

**Scheme 4:**
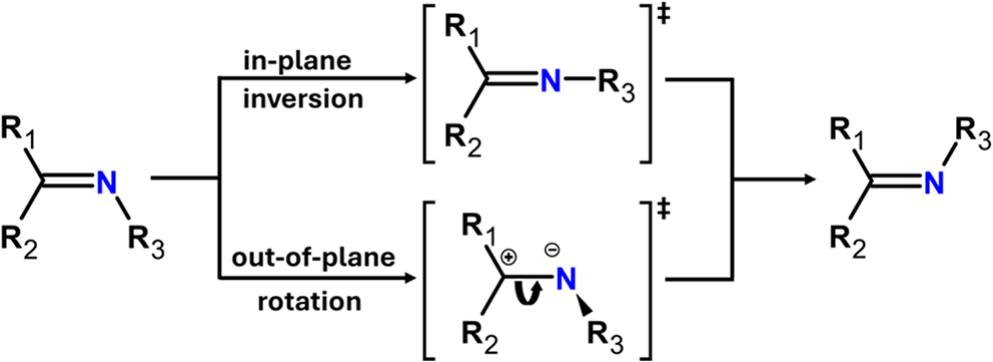
Mechanism of *Z*, *E* Isomerism for Hydrazones

First, the in-plane inversion mechanism of thermal switching was investigated by computing the transition state (***Z*** **-TS** ^**inv**^) for the reactions ***Z*** → ***E*** of compounds **1** and **2**, respectively (Figure 4). It resulted in Δ*G* ^‡^ ≈ 32 kcal·mol^–1^ for both compounds. Next, the intermediate (**Int**) is formed, which undergoes rotation to the *E*-isomer via the transition state (**Rot-TS**) with Δ*G* ^‡^ = 2.5 kcal·mol^–1^ for **1** and Δ*G* ^‡^ = 3.1 kcal·mol^–1^ for **2**.

**Figure 4:**
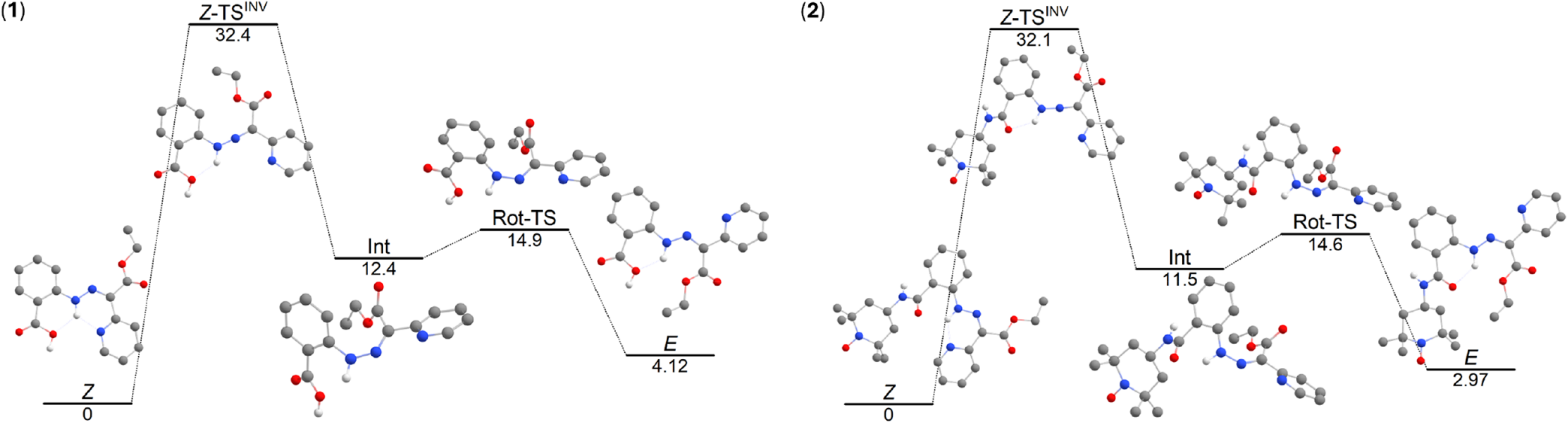
DFT-calculated Gibbs energy profile for the *Z* → *E* isomerization, comprising the in-plane inversion for **1** (left) and **2** (right). The values are in kcal·mol^–1^. The molecular structures are shown without hydrogen atoms attached to carbon atoms. Atom colors: dark gray (C), light gray (H), red (O), and blue (N).

Second, the mechanism based on the out-of-plane rotation was studied; Figure 5. It is well-known that such a mechanism can involve the transfer of a hydrogen atom. In such a case, the mechanism can be decomposed to following steps: (i) the tautomerization reaction involving the intramolecular proton transfer from azo-group to pyridine moiety (***Z*** → ***Z*** **-pyHb** for **1** and ***Z*** → ***Z*** **-pyH** for **2**), where also a respective transition state was identified (***Z*** **-TS** ^**H**^), (ii) the formation of the hydrogen bond between the hydrogen of the carboxylic group and the nitrogen of the azo group in the case of **1** (***Z-*** **pyHb** → ***Z*** **-pyH**), (iii) rotation of azo-group (***Z*** **-pyH** → ***E*** **-pyH**) and locating the respective transition state (**TS-pyH**), (iv) and finished by the intra/intermolecular hydrogen transfer, hence by the tautomerization (***E*** **-pyH** → ***E***). In the case of the first tautomerization (***Z*** → ***Z*** **-pyHb/** ***Z*** **-pyH**), the Gibbs energies of the transition states are comparable, 6.6 kcal·mol^–1^ for **1** and 5.4 kcal·mol^–1^ for **2**. The second activation Gibbs energy related to the formation of **TS-pyH** (Δ*G* ^‡ *Z*→*E*^) was found to be a bit smaller for **1**, with the value of 16.5 kcal·mol^–1^ in comparison to the value of 18.1 kcal·mol^–1^ for **2**. Moreover, the direct isomerization of **1** and **2** without considering proton transfer, according to the reaction *Z* → TS → *E*, was also evaluated for these compounds. This pathway involves rotation around the C=N double bond, which can lead to a transition state with a significant diradical character. To account for potential spin contamination and the possibility of a nonsinglet ground state during this rotation, Broken-Symmetry DFT (BS-DFT) calculations were employed.^60^ Indeed, BS-DFT provided lower energies of TS, which are reported in Figure 5 as **TS** ^**BS**^. Respective spin density plots are provided in Figure S19. It is evident that this mechanism provides slightly higher Δ*G* ^‡^ ≈ 40 kcal·mol^–1^ for both compounds than the in-plane inversion mechanism (Δ*G* ^‡^ ≈ 32 kcal·mol^–1^). To summarize, these calculations indicate that compounds **1** and **2** exhibit similar activation Gibbs energies (Δ*G* ^‡^) for both thermal isomerization mechanisms under consideration. Hence, the clue to the absence of photoisomerization of **2** is still missing.

**Figure 5:**
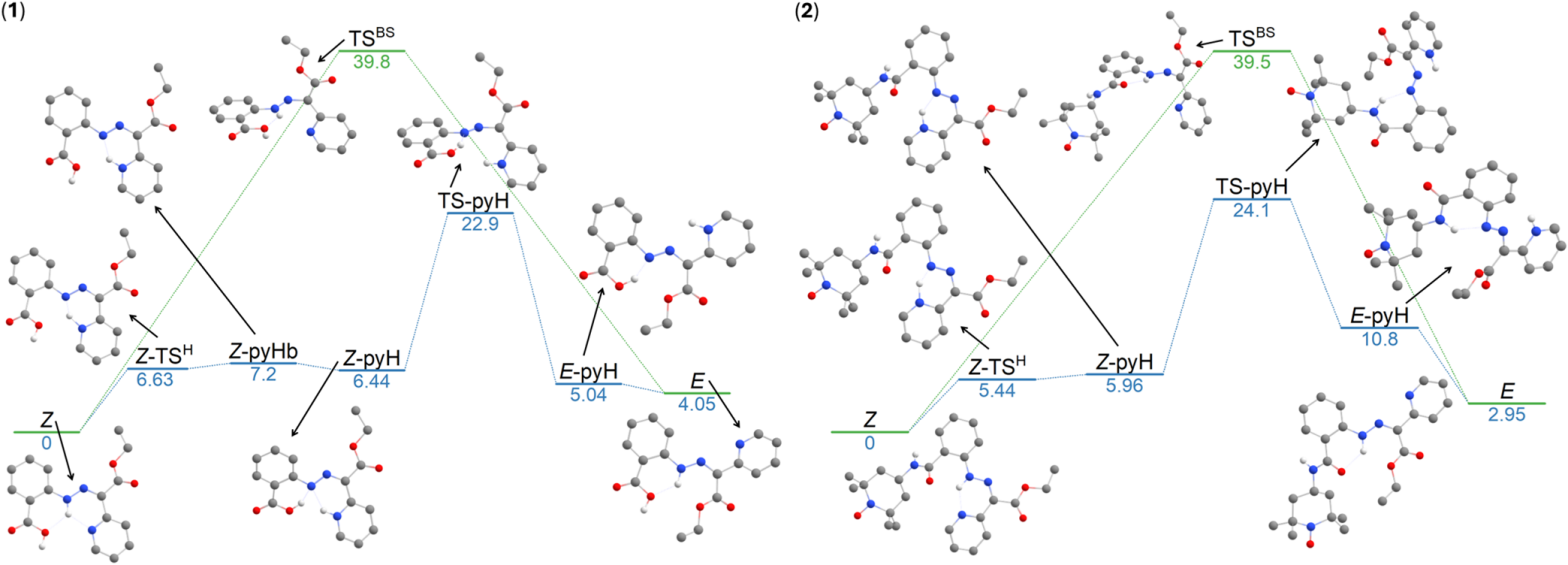
DFT-calculated Gibbs energy profile for the *Z* → *E* isomerization, comprising the out-of-plane rotation for **1** (left) and **2** (right). The values are in kcal·mol^–1^. The molecular structures are shown without hydrogen atoms attached to carbon atoms. Atom colors: dark gray (C), light gray (H), red (O), and blue (N).

Therefore, additional TD-DFT calculations were performed for **1** and **2**. In the case of **1**, the first S_0_ → S_1_ transition of ***Z*** has a vertical energy of 84.9 kcal·mol^–1^ and represents π → π* electron transfer as documented with natural transition orbitals (NTOs) in Figure 6.

**Figure 6:**
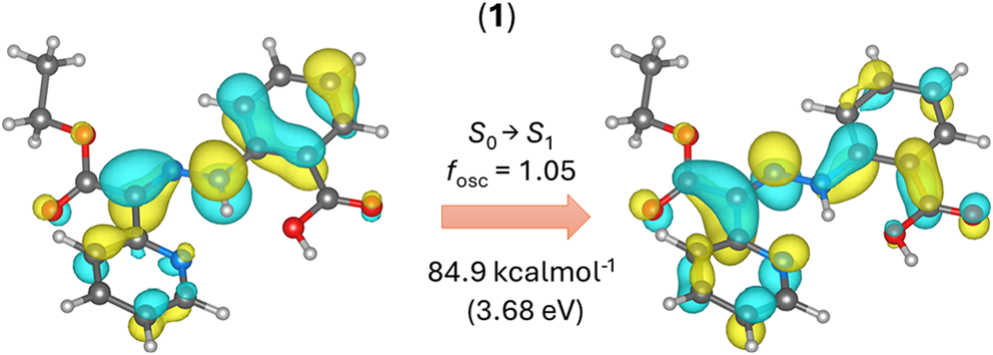
Results of TD-DFT calculations for **1-** ***Z*** isomer, showing the donor (left) and acceptor (right) NTO orbitals related to the S_0_ → S_1_ transition.

This transition contributes to weakening of the C=N double bond of the hydrazone moiety. However, similar calculations for **2-** ***Z*** isomer showed that the first D_0_ → D_1_ transition has an energy of 66.9 kcal·mol^–1^ and represents π → π* electron transfer within TEMPO moiety SOMO orbitals (Figure 7, top). Moreover, this first transition has a very small oscillation strength (*f*_osc_). Second excitation, D_0_ → D_2_, represents π → π* electron transfer within the hydrazone moiety, similarly to the S_0_ → S_1_ transition of **1**, and has a comparable energy of 86.1 kcal·mol^–1^ and the same *f*_osc_ (Figure 7, bottom).

**Figure 7:**
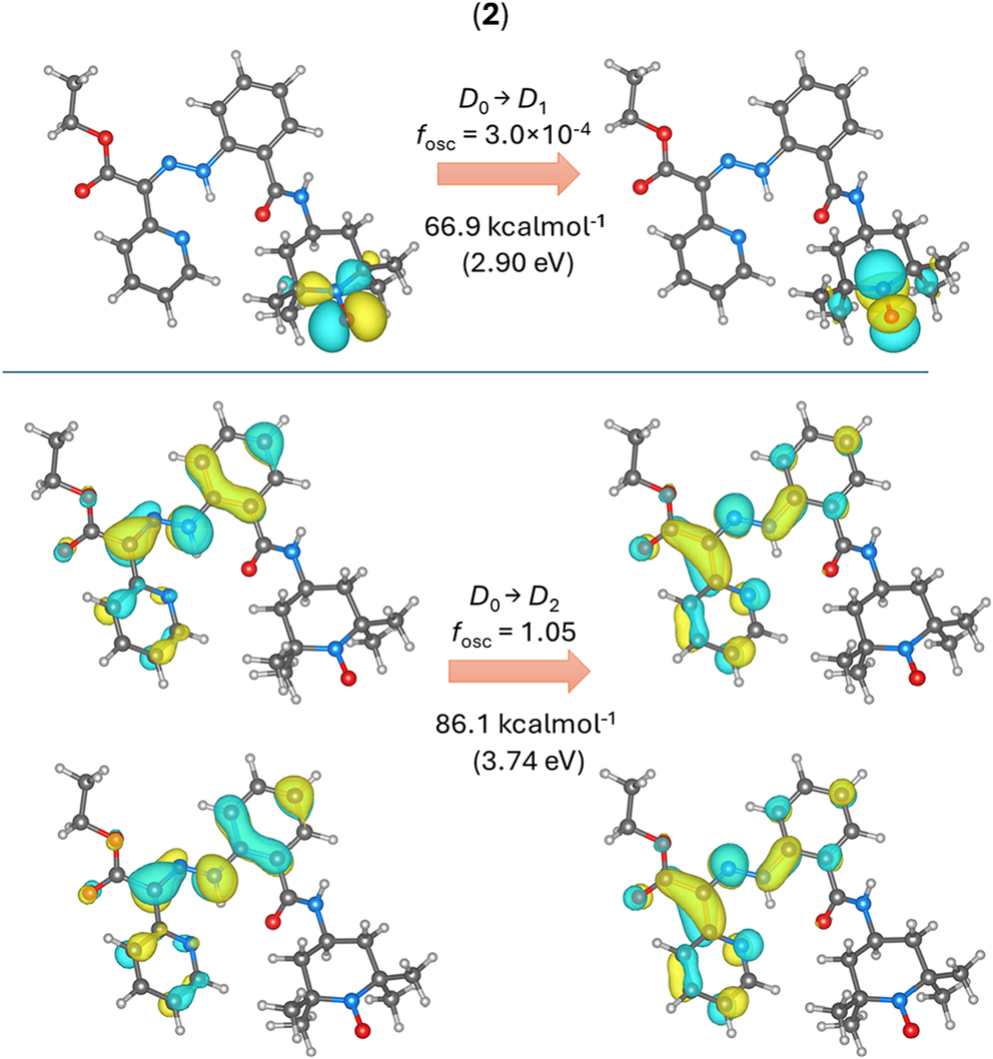
Results of TD-DFT calculations for the **2-** ***Z*** isomer, showing the donor (left) and acceptor (right) NTO orbitals related to the D_0_ → D_1_ transition (top) and the D_0_ → D_2_ transition (bottom).

It was also possible to optimize the molecular geometries of the excited state S_1_ of **1-** ***Z*** and D_2_ of **2-** ***Z***, as depicted in Figure 8. As these states are related to π → π* electron transfer within the hydrazone moiety, the rotation around the hydrazone bond was observed as expected, which resulted in dihedral angles of C–N–N–C ≈ 85° and elongation of the originally C=N bond and shortening of the N–N bond. Analogous results were obtained for the **1-** ***E*** and **2-** ***E*** isomers, as shown in Figure S20.

**Figure 8:**
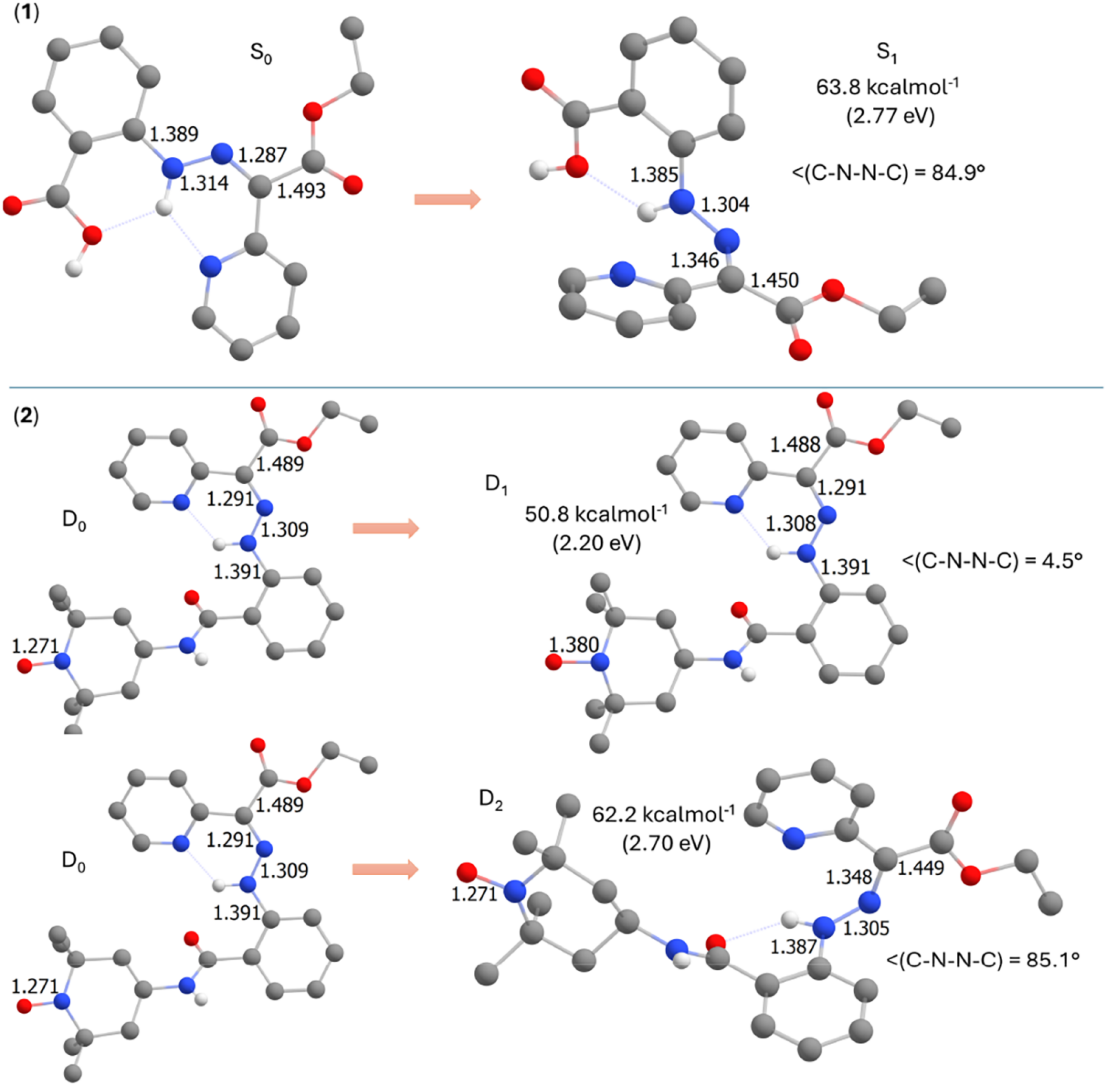
Results of TD-DFT calculations for the ***Z*** isomers of **1** and **2** are presented, comparing the molecular geometries of the ground states and optimized excited states (S_1_ for **1**, D_1_ and D_2_ for **2**). Selected geometrical parameters and the adiabatic energies of the excited states are also included.

These calculations suggest that the behaviors of **1** and **2** should be similar when exposed to light. The only difference is that compound **2** provides an excited state D_1_ related to the TEMPO group (Figures 7 and 8). As *f*_osc_ of this transition is very small, it is more likely that D_1_ can be populated through D_2_ → D_1_ internal conversion. Therefore, the conical intersection (CI) of D_2_ and D_1_ was searched for, and the results are shown in Figure 9. Please note that the N–O bond distance increased and the dihedral angle C–N–N–C decreased in comparison to the D_2_ geometry. The energy of CI is 74.4 kcal·mol^–1^ which is only 12.2 kcal·mol^–1^ energy difference to D_2_ state (62.2 kcal·mol^–1^, Figure 8). Thus, this energy barrier could be less than the energy barrier required for the photoisomerization reaction, providing a plausible explanation for quenching the photoisomerization process in **2**.

**Figure 9:**
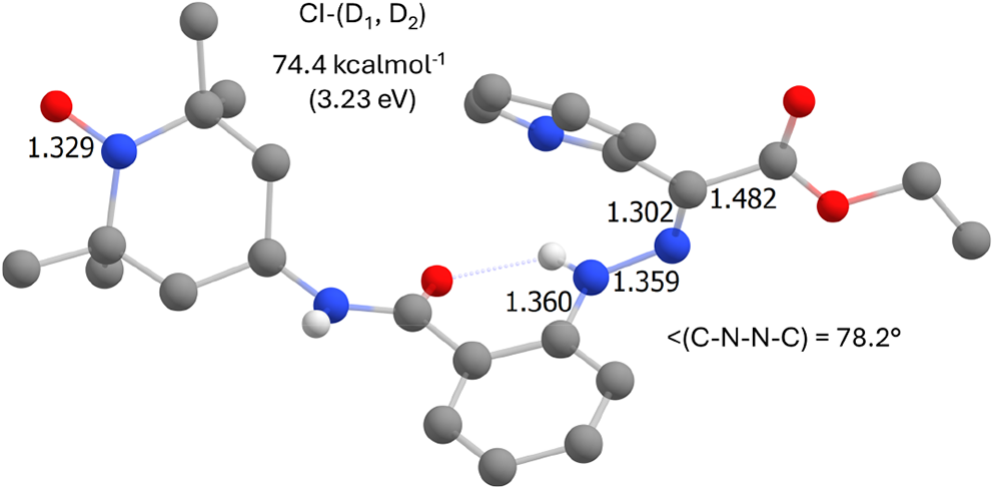
Results of TD-DFT calculations for the **2-** ***Z*** isomer, showing the molecular geometry of the conical intersection between two excited states D_1_ and D_2_. Selected geometrical parameters and the adiabatic energy of the CI-state are also included.

It must be noted that the NEB method was utilized for the search for the photoisomerization transition states within the S_1_ manifold between **1-** ***Z*** and **1-** ***E***, and within the D_2_ manifold between **2-** ***Z*** and **2-** ***E***, but such calculations did not converge. Moreover, having an open-shell molecule of **2** with a doublet ground state, we also applied the collinear spin-flip TD-DFT (SF-TD-DFT) method.^61^ However, it was found that geometry optimization did not follow the selected excited state, and additionally, analyzing the results is challenging due to the absence of NTOs for this method.

## Conclusions

A new nitroxide-substituted hydrazone switch (**2**) was successfully synthesized and characterized by ^1^H NMR, MS, UV–vis, EPR, and X-ray diffraction analysis. In the next step, we aimed to experimentally and theoretically investigate the switching properties. We demonstrated that the hydrazone precursor (**1**) undergoes partial photoisomerization (yielding 59% of the *Z*-isomer and 41% of the *E*-isomer) that is reversible. However, the attachment of the nitroxide moiety inhibits photoswitching in compound **2** under 420 nm irradiation. Even after 2 h of irradiation, no change in the *Z*/*E* isomer ratio was observed in the ^1^H NMR spectrum. Compound **2** showed considerable robustness against 420 nm irradiation, showing a minimal EPR signal intensity loss. In contrast, irradiation at 365 nm led to significant photobleaching. On the other hand, pH-induced switching (monitored by UV–vis) indicated reversible isomerization, which was supported by changes in τ*_c_* values extracted from EPR spectra.

The restricted photoisomerization of compound **2** was initially attributed mainly to strong intramolecular hydrogen bonding between the pyridine nitrogen and the N–H proton of the hydrazone moiety. Therefore, removing this hydrogen bond by replacing the pyridine ring with a phenyl ring may further facilitate photoisomerization.

QT-AIM analysis revealed stronger intramolecular hydrogen bonds in compound **2** in the solid state. Theoretical calculations at the DFT level showed similar properties of **1** and **2** for both in-plane inversion and out-of-plane rotation thermal isomerization mechanisms. Additional TD-DFT calculations revealed the existence of the S_1_ excited state of **1** and the D_2_ excited state of **2** related to π → π* electron transfer within the hydrazone moiety, resulting in weakening of the C=N bond and enabling rotation around the hydrazone bond. This should be the first step in the photoisomerization process. However, the presence of the D_1_ excited state in **2**, which is related to π → π* electron transfer of the TEMPO group, might be responsible for the internal conversion of D_2_ → D_1_. As the D_1_ excited state prolongs the N–O bond and does not affect the hydrazone bond, such an internal conversion can lead to quenching of the photoisomerization process. Therefore, further research should focus on radical-decorated hydrazones where the lowest excited state is on the hydrazone rather than on the radical group.

## Supplementary Material





## Data Availability

The data underlying this study are available in the published article and its Supporting Information.

## Notes

Characterization (^1^H, ^13^C­{^1^H} NMR, ESI-MS, UV–vis, X-band EPR, X-ray crystallographic data) of compounds **1** and **2**, switching analysis, and computational data, including *XYZ* coordinates (PDF)
ES-XYZ (ZIP)

## Abbreviations

BS-DFT: broken-symmetry density functional theory
CDI: 1, 1′-carbonyldiimidazole
DCM: dichloromethane
DNP: dynamic nuclear polarization
EPR: electron paramagnetic resonance spectroscopy
MS: mass spectrometry
MOST: molecular solar thermal energy storage
NEB: nudged elastic band method
NMR: nuclear magnetic resonance spectroscopy
PELDOR: pulsed electron–electron double resonance spectroscopy
QT-AIM: quantum theory of atoms in molecules
SF-TD-DFT: spin-flip time-dependent density functional theory
TD-DFT: time-dependent density functional theory
TEMPO: (2,2,6,6-Tetramethylpiperidin-1-yl)­oxyl
TFA: trifluoroacetic acid
TLC: thin layer chromatography
TS: transition state
