# Supplementary material for: Novel Nitroxide-Substituted Hydrazone Switch: Experimental and Theoretical Insights into Photoswitching Behavior
Source: ACS Org Inorg Au. 2025 Dec 3;6(1):64–75. doi: 10.1021/acsorginorgau.5c00068 (PMC12879170; doi:10.1021/acsorginorgau.5c00068)
Supplement: Supplementary file 1 [file gg5c00068_si_001.pdf]

A Novel Nitroxide-Substituted Hydrazone Switch: Experimental and Theoretical Insights into Photoswitching Behavior

Lucie Kotásková,[a*] Ivan Nemec,[a,b*] Radovan Herchel,[b] Vinicius T. Santana,[a] Petr Neugebauer[a]

[a]Central European Institute of Technology, Brno University of Technology, Purkyňova 656/123, 61200 Brno, Czech Republic

[b]Department of Inorganic Chemistry, Faculty of Science, Palacký University, 17 listopadu 12, 77900 Olomouc, Czech Republic

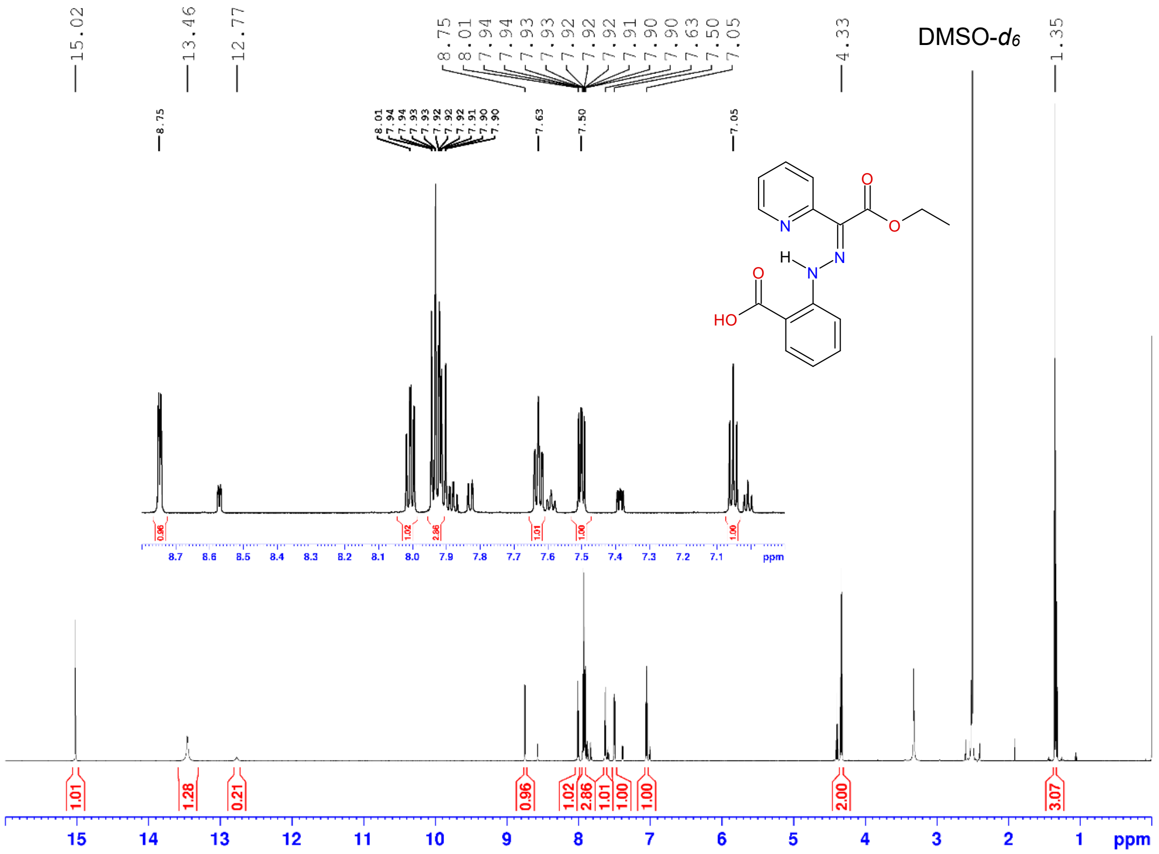

Fig. S1. ^{1}H NMR spectrum of compound **1** (700 MHz, DMSO-d_6, 298 K).

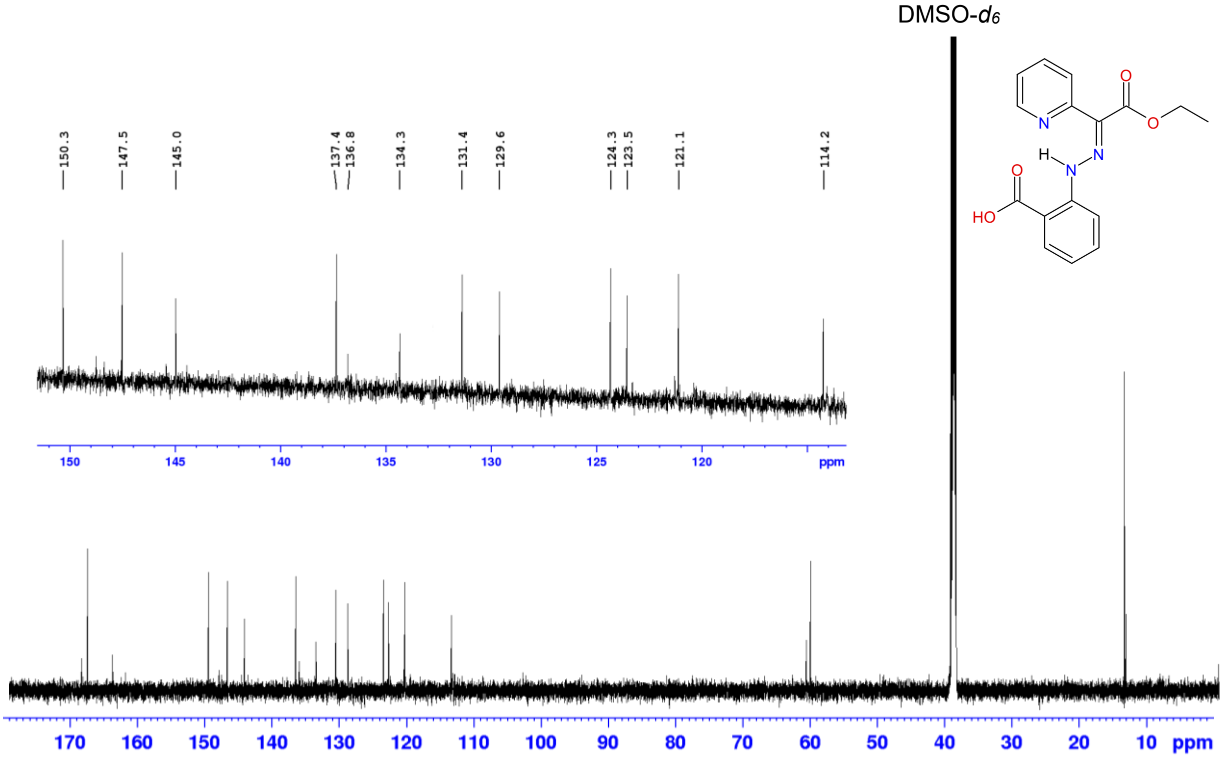

Fig. S2. ^{13}C{^{1}H} NMR spectrum of compound **1** (176 MHz, DMSO-d_6, 298 K).

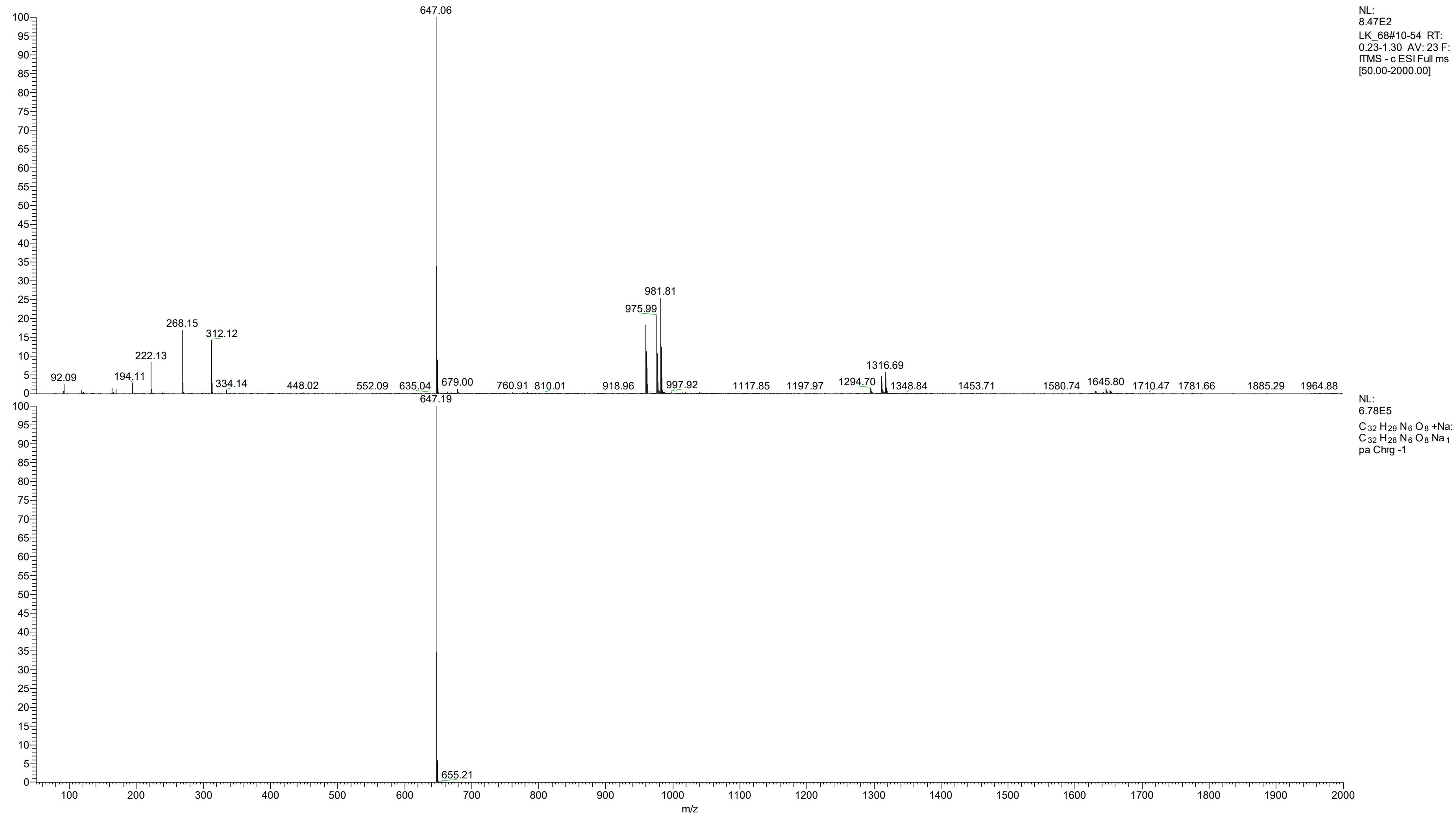

Fig. S3. MS spectrum of **1** ($C_{16}H_{15}N_3O_4$) in MeOH [2×(M–H)$^{\cdot}$+Na]$^-$ ion, top) and simulated spectrum (bottom).

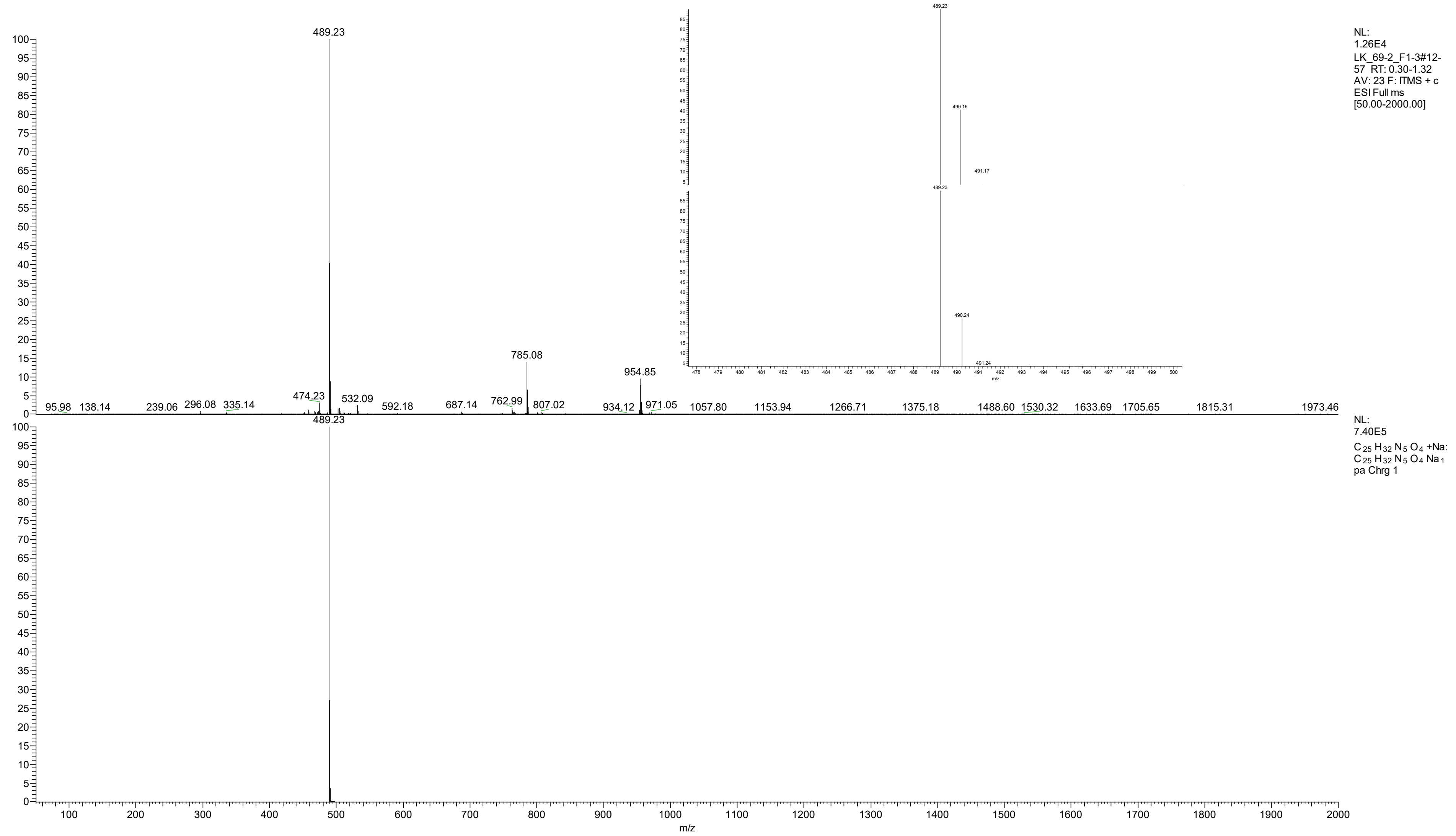

Fig. S4. MS spectrum of **2** in MeOH ([$C_{25}H_{32}N_5O_4^{\cdot}$+Na]$^+$ ion, top) and simulated spectrum (bottom).

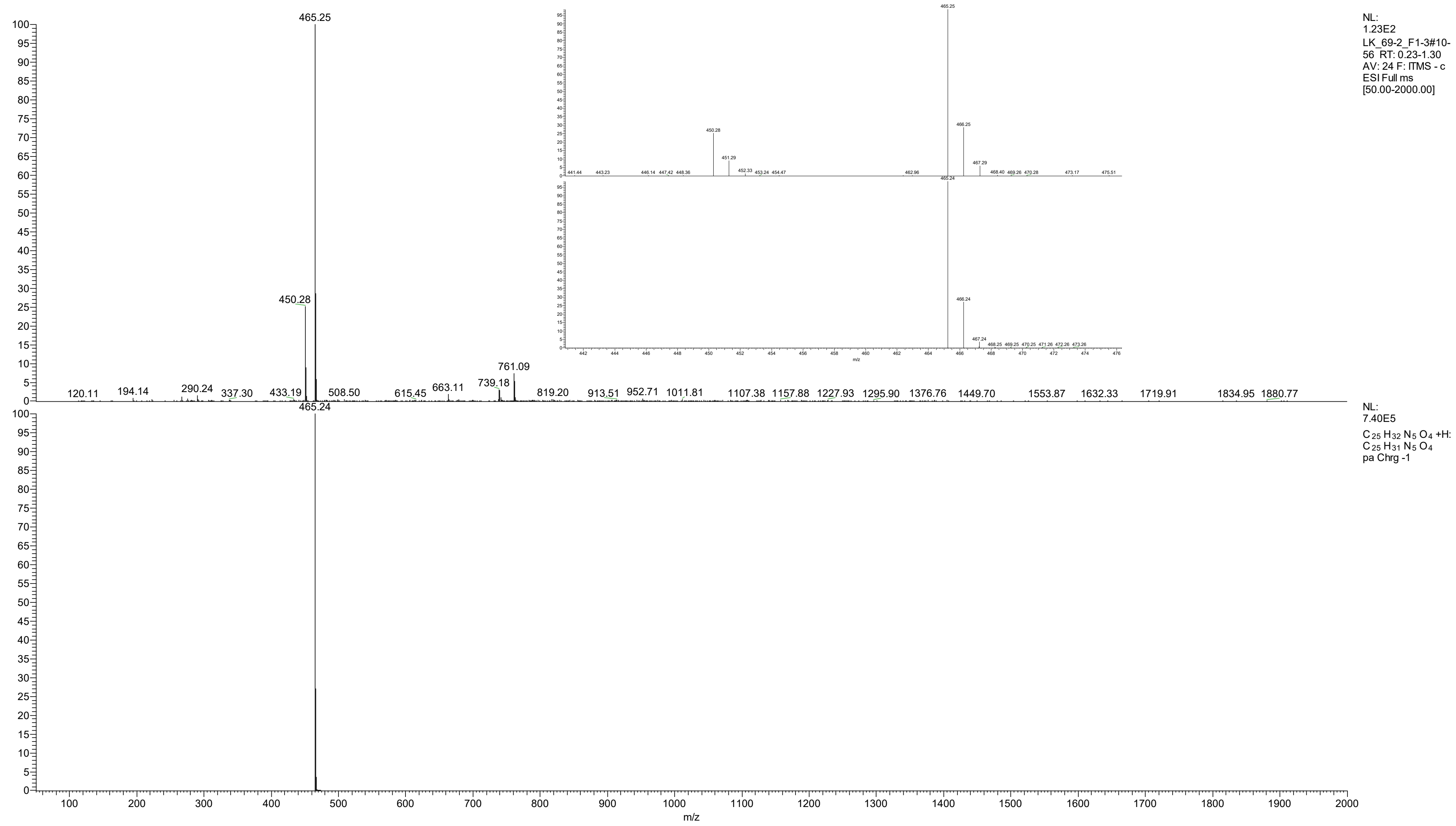

Fig. S5. MS spectrum of **2** in MeOH ([$C_{25}H_{32}N_5O_4^{\cdot}$–H]$^{-}$ ion, top) and simulated spectrum (bottom).

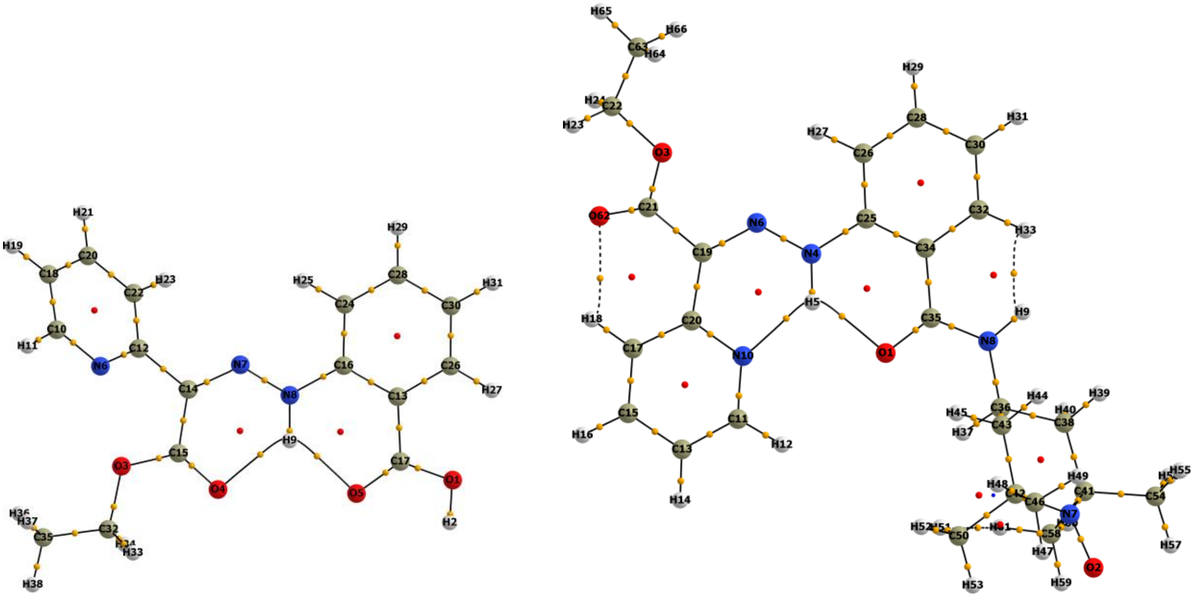

Fig. S6. QT-AIM molecular graphs for **1** (left) and **2** (right). The (3,-1) bond critical points are shown as yellow dots, while the (3,+1) ring critical points are represented as red dots.

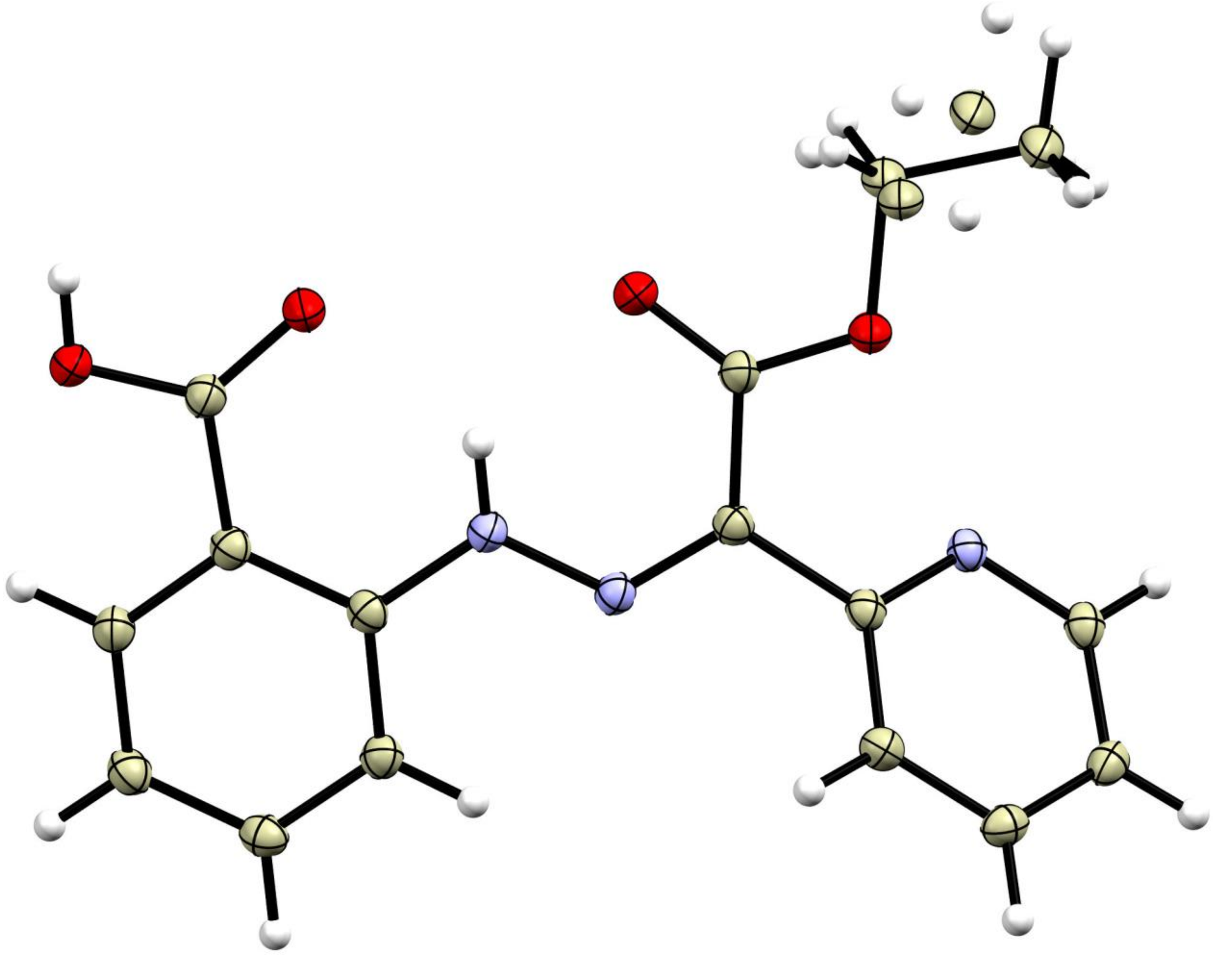

Fig. S7. Thermal ellipsoid plot of compound **1** at the 30% probability level.

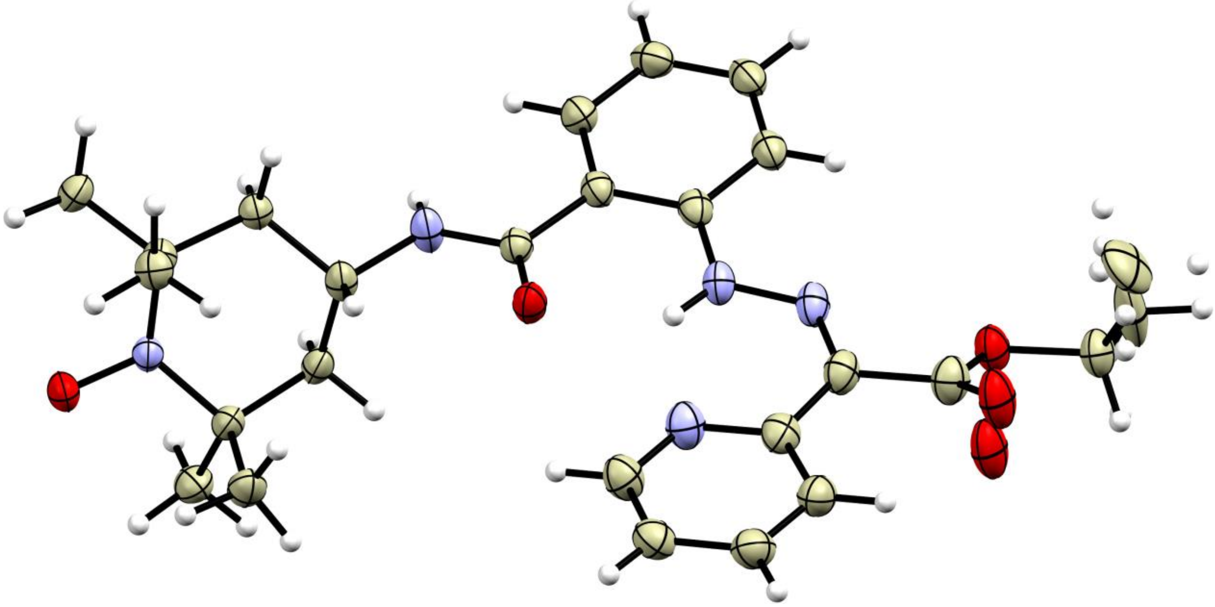

Fig. S8. Thermal ellipsoid plot of compound **2** at the 30% probability level.

Photoswitching of compounds **1** and **2**

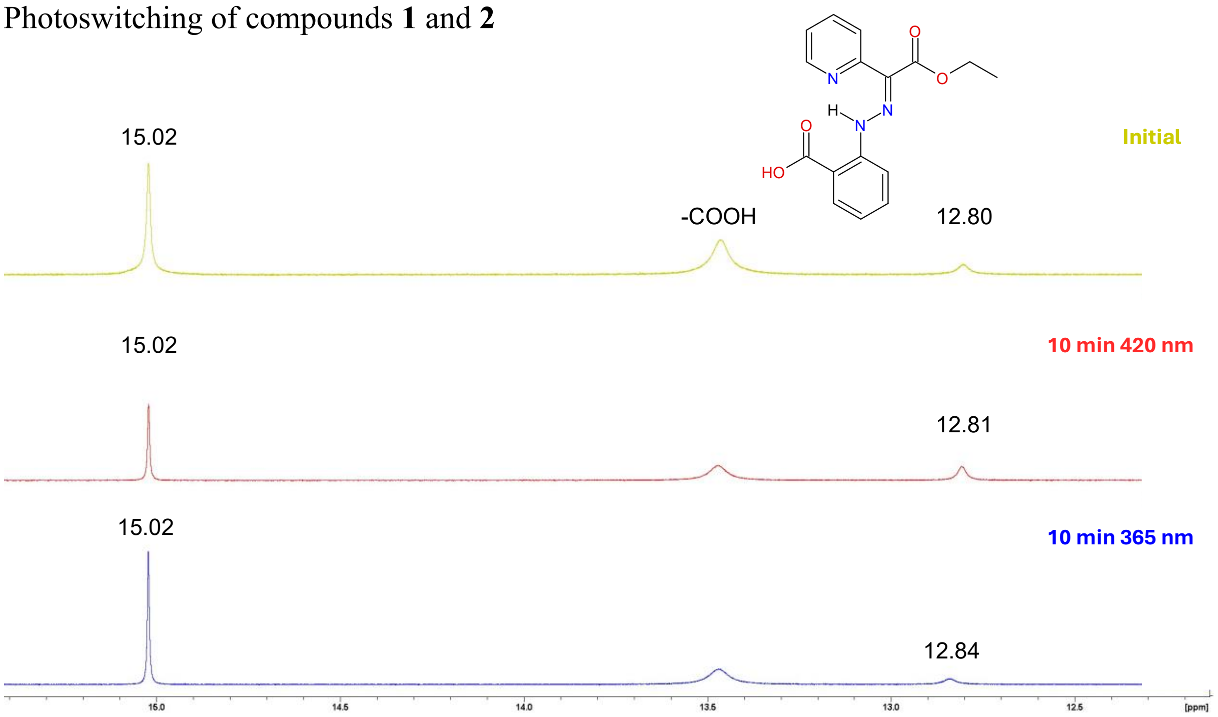

Fig. S9. Comparison of N–*H* shifts (in ppm) in ^{1}H NMR spectra of compound **1** before (yellow) and after irradiation at 420 nm (red) and 365 nm (blue) for 10 minutes. Spectra are shown in the range between 12 and 14.5 ppm. DMSO-d_6, 500 MHz, 298 K.

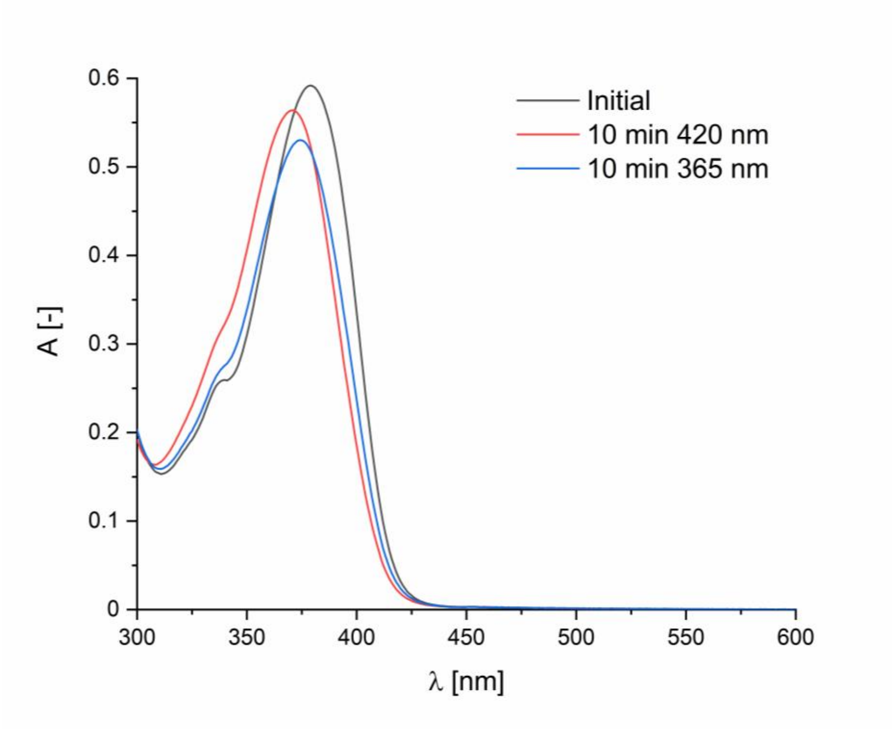

Fig. S10. UV-Vis spectrum of compound **1** (1.93×10^{-5} M, toluene) before and after photoswitching at 420 nm and 365 nm for 10 minutes.

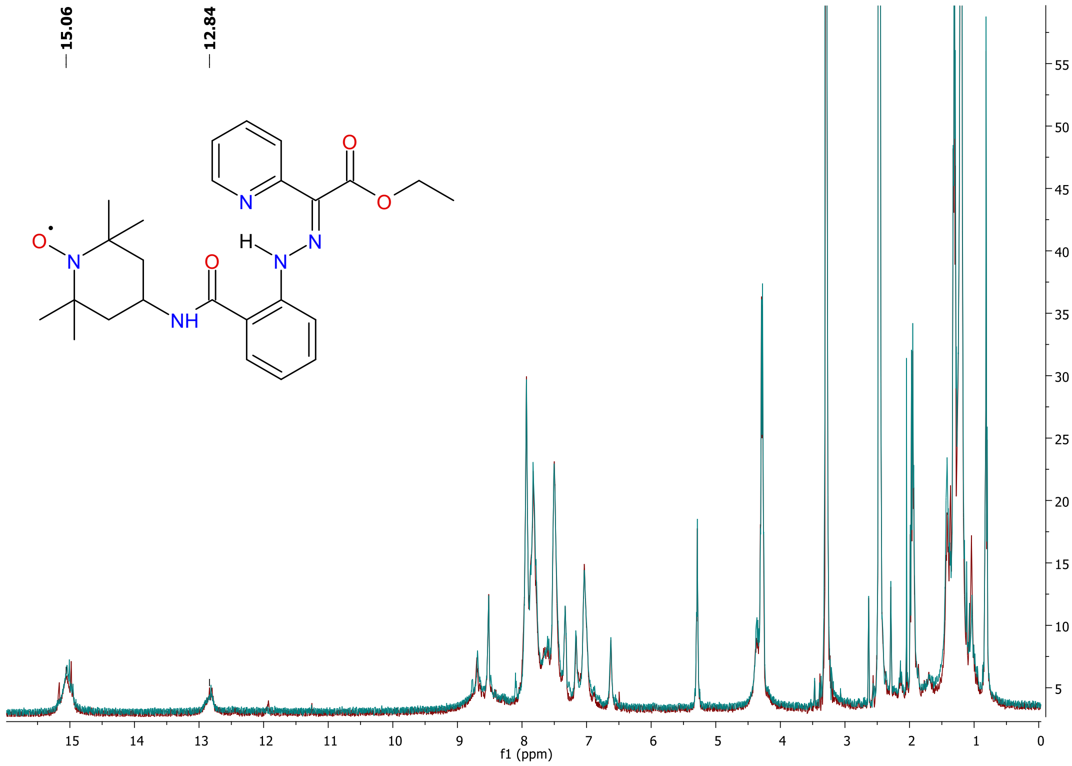

Fig. S11. Comparison of ^{1}H NMR spectra of compound **2** before (green) and after irradiation at 420 nm (red) for 2 hours (DMSO-d_6, 400 MHz, 298 K).

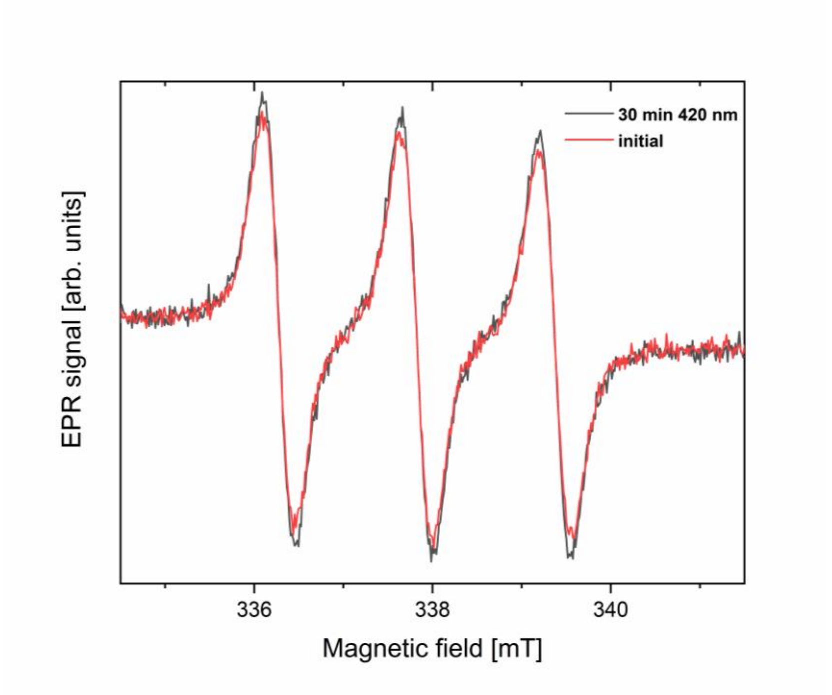

Fig. S12. X-band EPR spectrum of compound **2** (RT, toluene) before and after irradiation at 420 nm wavelength for 30 minutes.

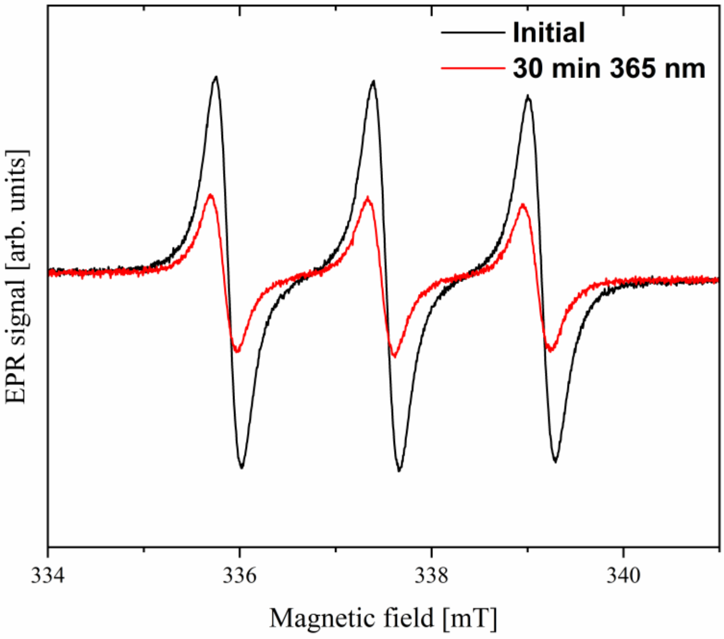

Fig. S13. X-band EPR spectrum of compound **2** (RT, toluene) before and after irradiation at 365 nm wavelength for 30 minutes.

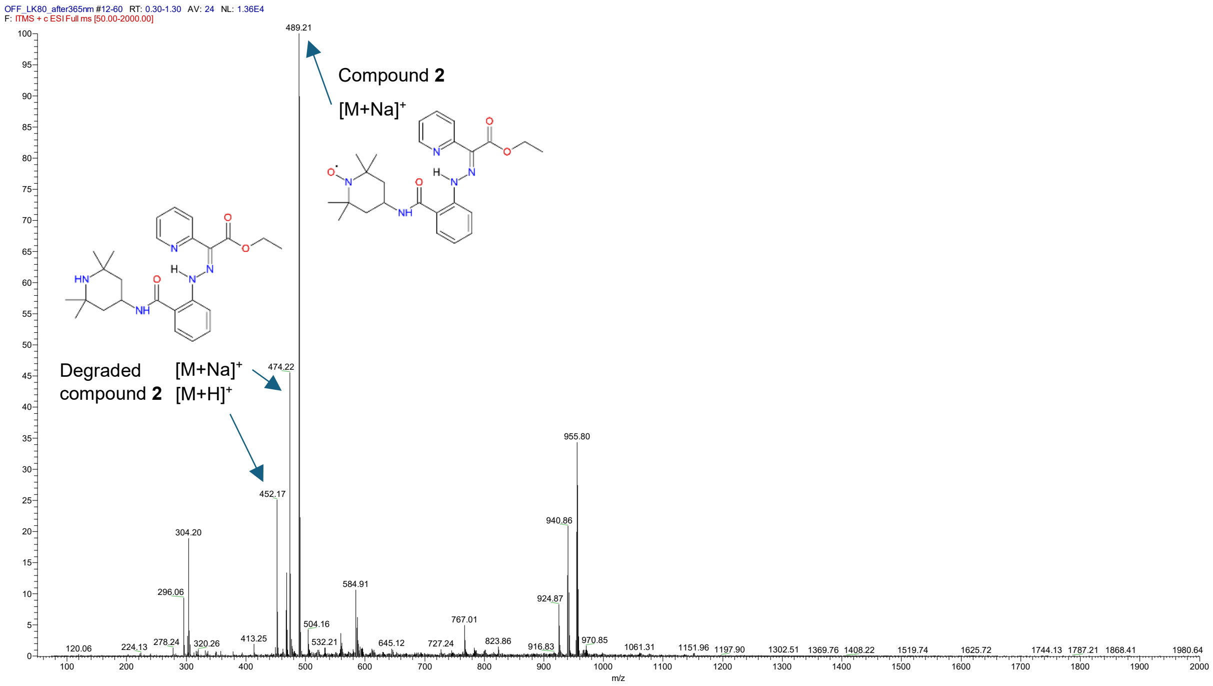

Fig. S14. MS spectrum of **2** upon irradiating at 365 nm for 30 minutes (measured in MeOH). The degraded analogue and its adducts formed upon irradiating are highlighted.

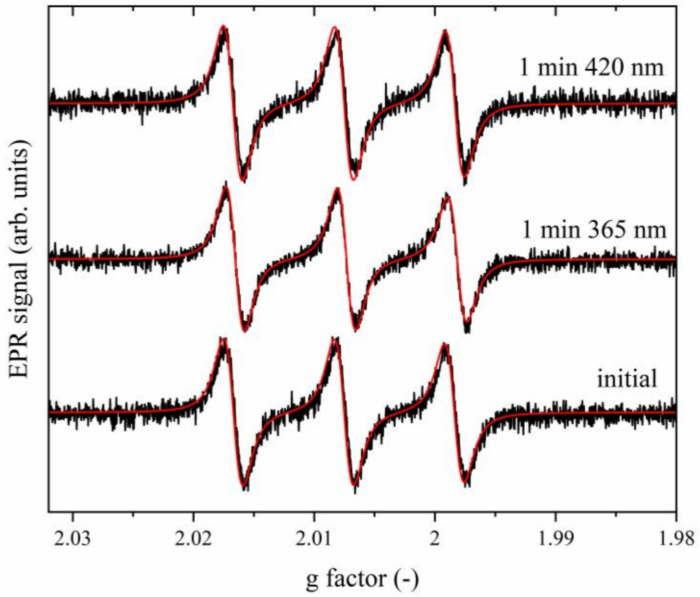

Fig. S15. X-band EPR spectra of compound **2** before and after photoswitching at 365 nm and 420 nm. 45 μM, toluene, 293 K.

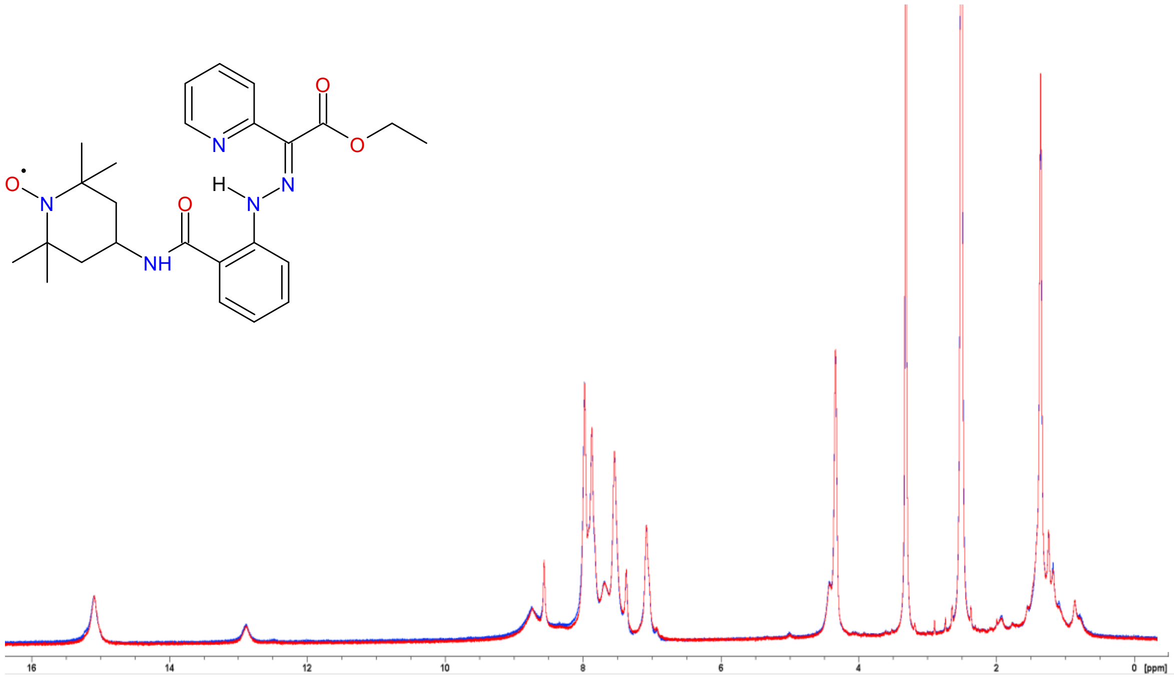

Fig. S16. Comparison of ^{1}H NMR spectra of compound **2** before (blue) and after irradiation at 365 nm (red) for 2 hours (DMSO-d_6, 500 MHz, 298 K).

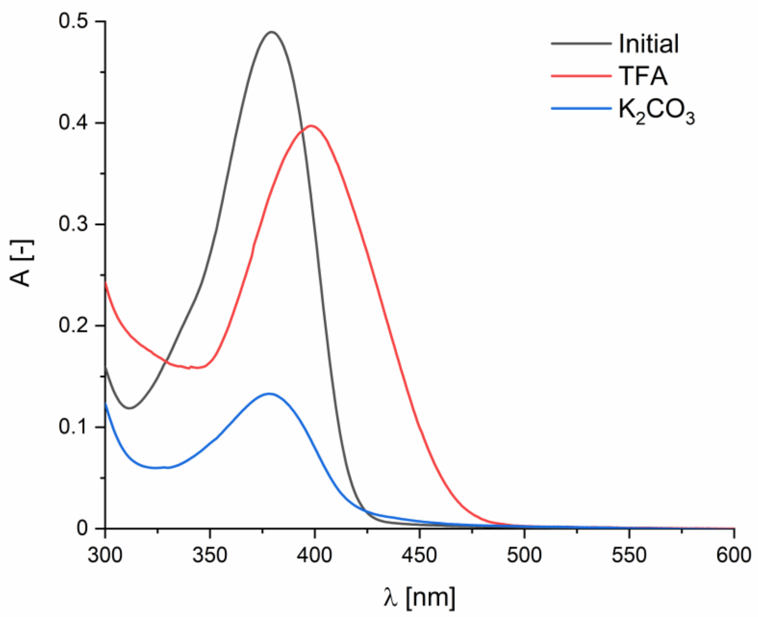

Fig. S17. UV-Vis spectrum of compound **1** (1.93×10^{-5} M, toluene) before and after pH switching using trifluoroacetic acid (100 eq.) and filtering through the plug of K_2CO_3.

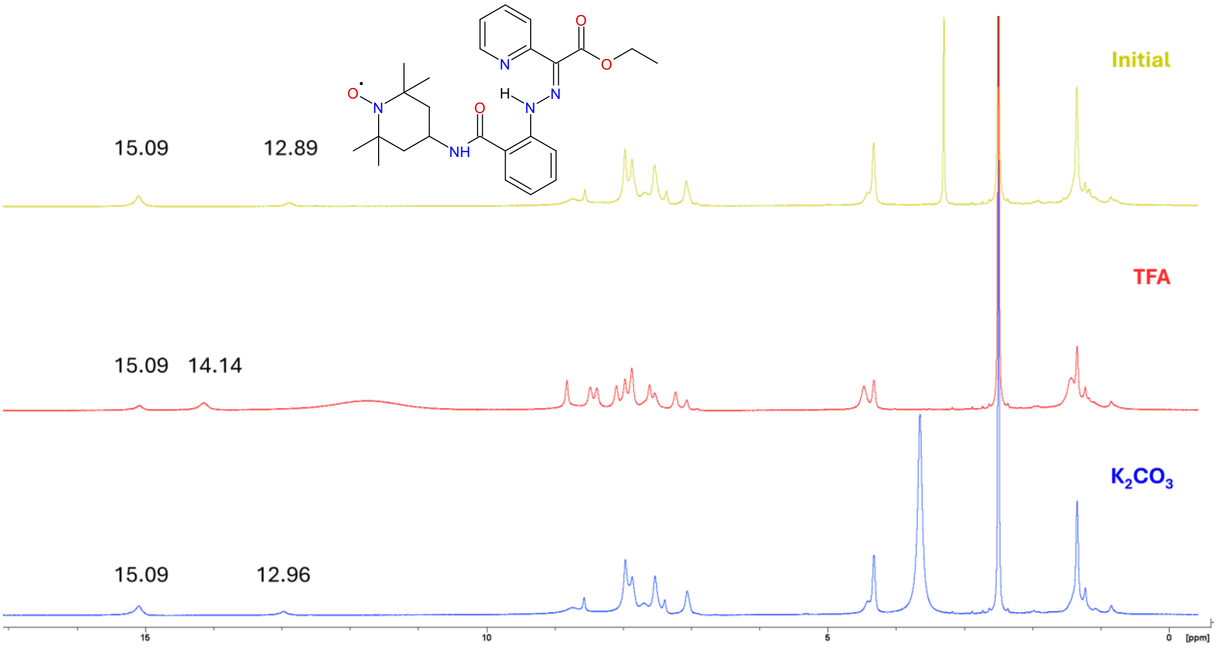

Fig. S18. Comparison of ^{1}H NMR spectra of compound **2** before and after pH switching using trifluoroacetic acid (2.6 eq.) and filtering through the plug of K_2CO_3 (DMSO-d_6, 500 MHz, 298 K).

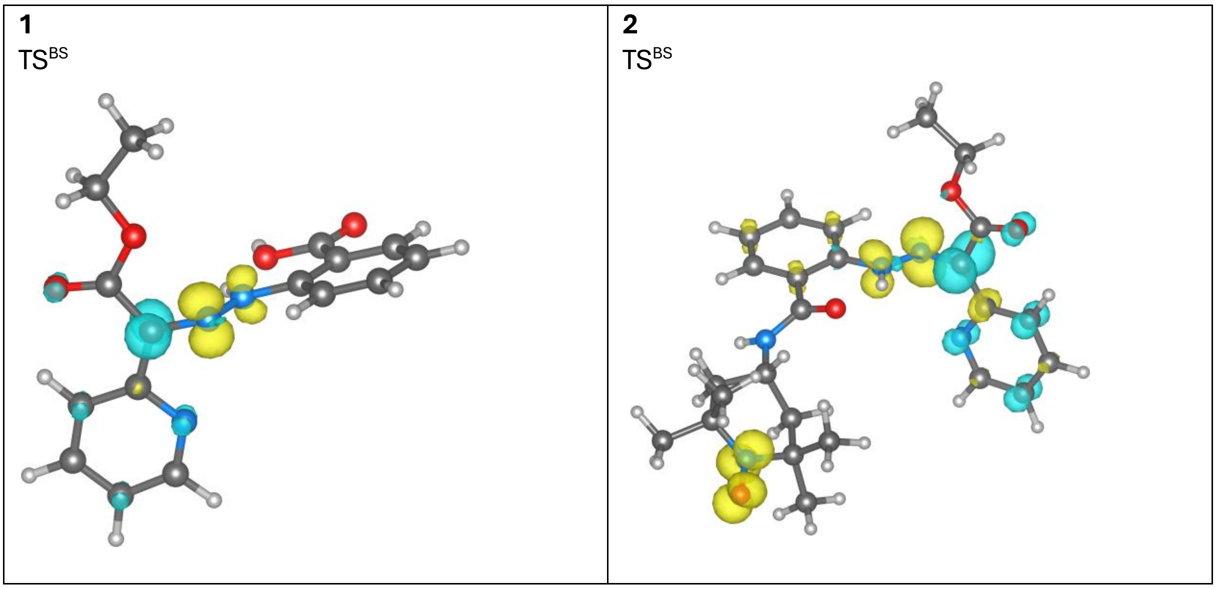

Fig. S19. The molecular geometries of TS^{BS} states for **1** and **2** related to **Figure 5** showing the spin density plots.

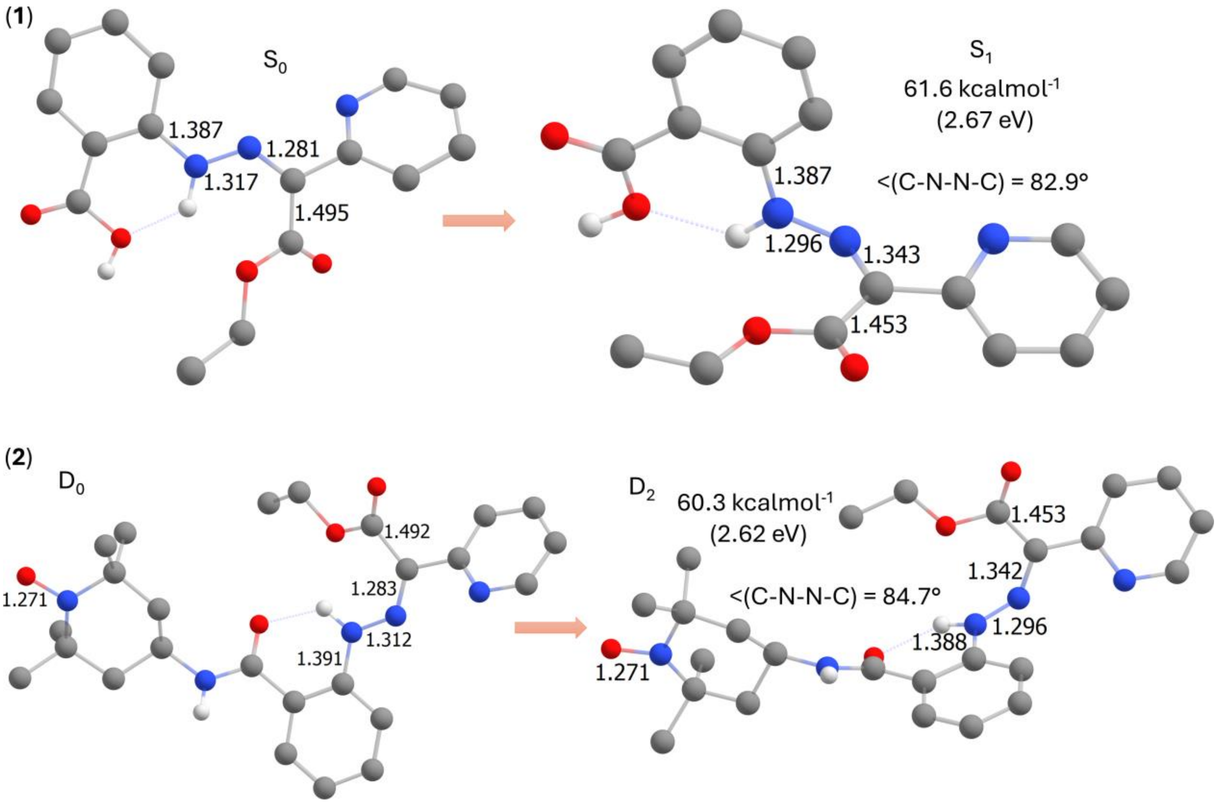

Fig. S20. The results of TD-DFT calculations for the ***E*** isomers of **1** and **2** are presented, comparing the molecular geometries of the ground states and optimized excited states (S_1 for **1**, D_2 for **2**). Selected geometrical parameters and the adiabatic energies of the excited states are also included.

Table S1. Crystal data and structure refinement for **1** and **2**.

	**1**	**2**
Formula	$C_{16}H_{15}N_3O_4$	$C_{25}H_{32}N_5O_4$
M_r	313.31	466.55
Crystal system	monoclinic	monoclinic
Space group	$P2_1/n$	$P2_1/c$
T/K	100.00(10)	90.0(3)
a (Å)	6.82750(10)	15.5288(5)
b (Å)	15.0049(2)	11.1962(3)
c (Å)	14.5419(2)	15.2198(4)
α (°)	90	90
β (°)	94.8060(10)	110.460(3)
γ (°)	90	90
V (Å^3)	1484.52(4)	2479.24(13)
Z	4	4
λ (Å), Cu Kα	1.54184	1.54184
D_{calc} (g·cm^{-3})	1.402	1.250
μ (mm^{-1})	0.858	0.702
F (000)	656.0	996.0
Reflections collected, R_{int},	16367, 0.0526	13626, 0.0247
Data/restraints/parameters	2871/3/218	4514/7/326
Goodness-of-fit on F^2	1.032	1.045
Final R indices [$I > 2\sigma(I)$]	$R_1 = 0.0402$<br>$wR_2 = 0.0402$	$R_1 = 0.0517$<br>$wR_2 = 0.1476$
Final R indices (all data)	$R_1 = 0.0419$<br>$wR_2 = 0.1125$	$R_1 = 0.0657$<br>$wR_2 = 0.1579$
Larg. d. peak/hole / e Å^{-3}	0.34/-0.22	0.45/-0.22
CCDC no.	2425051	2425050

Table S2. Selected parameters obtained from X-band EPR measurements of compound **2** (45 mM, in toluene, 298 K) before and after pH and photoswitching. Microwave power: 10 mW, modulation amplitude: 0.5 G, modulation frequency: 100 kHz, sweep width: 100 G, sweep time: 50.04 s.

		Compound **2**				
Selected EPR parameters		Initial	TFA	K_2CO_3	1 min 365 nm	1 min 420 nm
g-factor	g_x	2.0108	2.0104	2.0108	2.0106	2.0108
	g_y	2.0078	2.0074	2.0078	2.0076	2.0078
	g_z	2.0038	2.0034	2.0038	2.0036	2.0038
Coupling constant [MHz]	A_x	18.2	18.4	17.3	19.8	17.5
	A_y	22.1	23.3	25.0	23.8	23.9
	A_z	91.6	97.7	90.0	89	91.0
Rotational correlation time τ_c [s]		4.05×10^{-11}	1.92×10^{-10}	2.12×10^{-11}	1.08×10^{-10}	4.11×10^{-11}
Resonance frequency [GHz]		9.638	9.646	9.634	9.646	9.629

Table S3. Topological and energetic properties of $\rho(\mathbf{r})$ calculated for **1** at the selected (3, -1) critical points. All values are provided in atomic units (a. u.) unless stated otherwise. Atom labelling is shown in Fig. S6.

Atoms		ε	$G(\mathbf{r})$	$H(\mathbf{r})$	$V(\mathbf{r})$	E_{int}/kcal.mol^{-1}
O4 - H9	+0.099697	0.090337	0.024879	+0.024924	-0.024834	-7.79
O5 - H9	+0.101817	0.050796	0.025813	+0.025454	-0.026171	-8.21

Table S4. Topological and energetic properties of $\rho(\mathbf{r})$ calculated for **2** at the selected (3, -1) critical points. All values are provided in atomic units (a. u.) unless stated otherwise. Atom labelling is shown in Fig. S6.

Atoms		ε	$G(\mathbf{r})$	$H(\mathbf{r})$	$V(\mathbf{r})$	E_{int}/kcal.mol^{-1}
O1 - H5	+0.109565	0.091034	0.028211	+0.027391	-0.02903	-9.11
H5 - N10	+0.106492	0.048053	0.029556	+0.026623	-0.03249	-10.19
